# Discovering the inhibition of YAP/TEAD Hippo signaling pathway via new pyrazolone derivatives: synthesis, molecular docking and biological investigations

**DOI:** 10.1038/s41598-024-79992-x

**Published:** 2024-11-21

**Authors:** Ahmed A. Noser, Maha M. Salem, Mohamed H. Baren, Adel I. Selim, Esraa M. ElSafty

**Affiliations:** 1 Organic Chemistry, Chemistry Department, Faculty of Science, Tanta University, Tanta, 31527 Egypt; 2 Biochemistry Division, Chemistry Department, Faculty of Science, Tanta University, Tanta, 31527 Egypt

**Keywords:** Pyrazolone, Molecular docking, Antioxidant, Hippo signaling pathway, ADMET, Cancer, Chemical biology

## Abstract

Heterocyclic compounds play a crucial role in the drug discovery process and development due to their significant presence and importance. Here, we report a comprehensive analysis of new pyrazolone derivatives, prepared according to a clear-cut, uncomplicated procedure. The pyrazolone derivatives were thoroughly characterized using various methods, such as elemental analysis, NMR, and FT-IR. The molecular docking interactions between the new pyrazolone derivatives and YAP/TEAD target protein observed that compound **4** had the top-ranked binding energy towards YAP/TEAD with a value equal to − 9.670 kcal/mol and this theoretically proves its inhibitory efficacy against YAP/TEAD Hippo signaling pathway. Besides, compound **4** showed the best IC_50_ against HCT-116, HepG-2, and MCF-7 (*in-vitro*) with IC_50_ 7.67 ± 0.5, 5.85 ± 0.4, and 6.97 ± 0.5 μM, respectively which confirmed our results towards suppressing YAP/TEAD protein (*in-silico*) compared with the IC_50_ of Sorafenib (SOR) reference chemotherapeutic drug 5.47 ± 0.3, 9.18 ± 0.6 and 7.26 ± 0.3 μM, respectively. Also, compound **4** showed no cytotoxic effects against human lung fibroblast normal cell line (WI-38) and its pharmacokinetics were elucidated theoretically using ADMET compared with SOR which observed highly toxic effects on normal cells with IC_50_ equal to 20.27 ± 0.45 μM. Additionally, compound **4** clarified a powerful antioxidant scavenging activity against DPPH free radicals (*in-vitro*). Conclusively, newly synthesized pyrazolone derivative **4** could be used as anticancer candidate via inhibiting the YAP/TEAD mediated Hippo signaling pathway.

## Introduction

Cancer is the most common disease-related death worldwide. Now, targeted therapy, surgery, and chemotherapy are the main axis for cancer treatment. The effectiveness of cancer therapy has been greatly enhanced by targeted therapy, which has become a common treatment for cancer patients^1^. The preceding perspective states that the regulation of cell proliferation and death by the Hippo signaling target pathway is essential for controlling organ growth and suppressing tumors^2^. The Hippo pathway is an attractive therapeutic target because its modulation has a major impact on patient prognosis and chemotherapeutic drug resistance^3^.

In both Drosophila and mammals, the kinase cascade known as the main Hippo pathway is well-established^4^. Through binding to 14-3-3 proteins, the Hippo pathway promotes the cytoplasmic retention and degradation of the yes-associated protein (YAP) and its paralog TAZ, (the transcriptional coactivator with PDZ-binding motif,) via phosphorylating and activating its core kinase cascade, which includes MST1/2 and LATS l/2^5^. When the Hippo pathway is suppressed either physiologically or pathologically, YAP/TAZ is dephosphorylated and then translocated into the nucleus, where it interacts with transcription factors belonging to the TEA domain family of growth-promoting proteins (TEAD1-4). Connective tissue growth factor (CTGF) and cysteine-rich angiogenic inducer 61 (CYR61), two downstream genes involved in cell migration and angiogenesis, are transcriptionally and translationally induced by the YAP–TEAD transcriptional complex when they bind to DNA via the TEAD DNA-binding domain^6^. It was possible to effectively repurpose verteporfin, an FDA-approved medication that is used as a photosensitizer in photodynamic therapy of macular degeneration, as a YAP-TEAD inhibitor^7^ as it’s unclear how specific it is to YAP-TEAD. Thus, there is a pressing need to identify and create novel YAP-TEAD-mediated Hippo signaling pathway-targeting compounds with high efficacy and minimal side effects.

Chalcones are α, β-unsaturated ketones consisting of two aromatic rings, they have a wide range of pharmacological and biological actions, making them intriguing synthons and bioactive scaffolds with significant therapeutic potential. Their substantial biological actions, including antibacterial, antioxidant, antitubercular, anticancer, and antileishmanial properties, are widely acknowledged^8–11^. Chalcones are useful in the synthesis of several heterocyclic compounds with potent biological activity, as it makes it easier to synthesize pyrimidinone derivatives by reacting with urea or thiourea^12^, pyrazoline derivatives by reacting with hydrazine and its derivatives^13^, pyridine derivatives through the reaction with ethylcyanoacetate^14^ and pyran derivatives through the reaction with malononitrile and piperidine^15,16^.

**Fig. 1:**
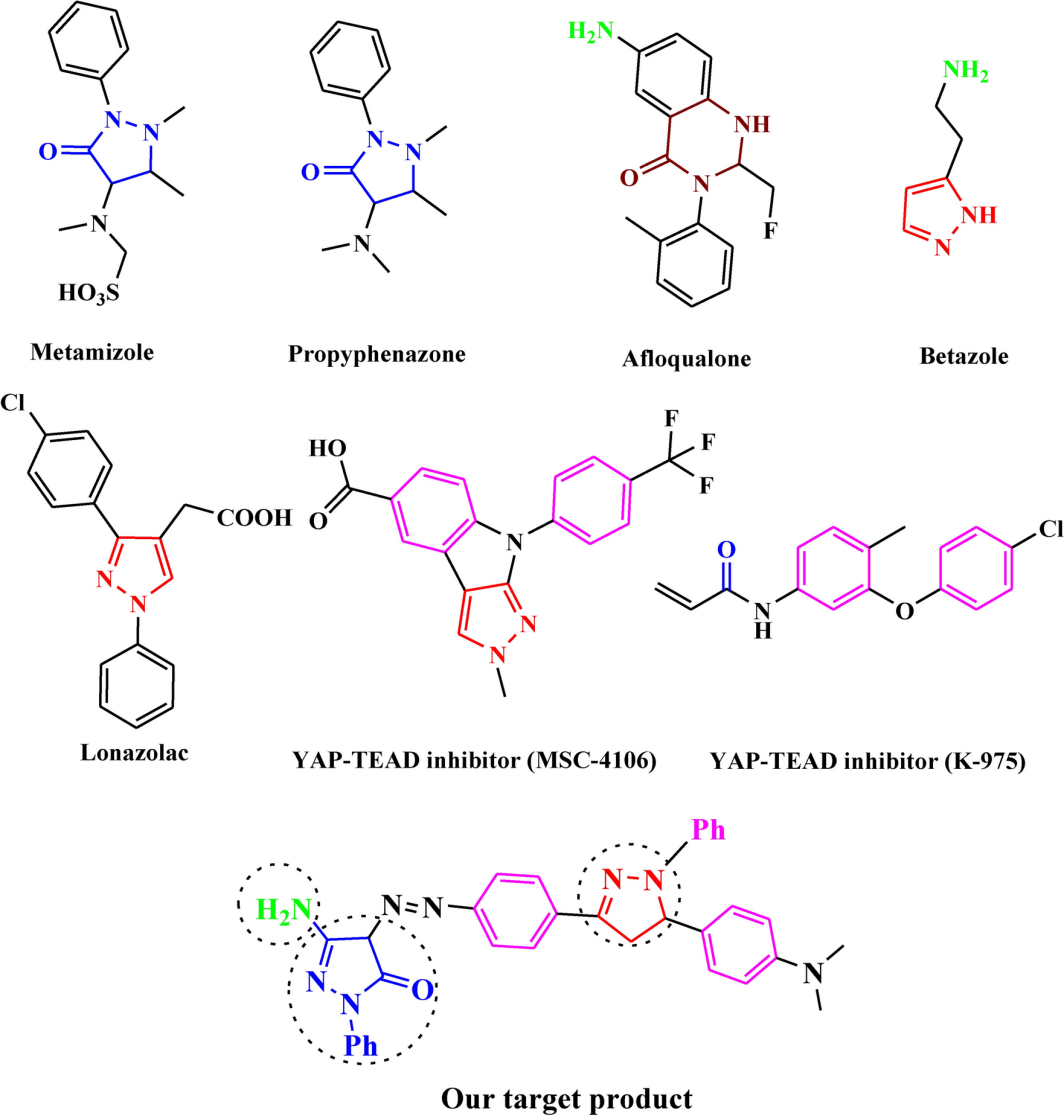
Marketed anti-cancer drugs containing pyrazolone moiety.

Skeletal pyrazolone derivatives are rich in physiologically significant compounds with a wide range of biological and pharmacological properties, such as antimicrobial, antitumor, anti-inflammatory, and antioxidant.^17–20^ The pyrazolone derivatives have a wide range of biological activities due to the strong aromaticity of the ring and the presence of heteroatoms, which directly block the YAP/TEAD Hippo signaling pathway. As illustrated in Fig 1, MSC-4106^21^, and K-975^22^ are approved drugs that directly inhibited the YAP-TEAD Hippo signaling pathway due to aromaticity and presence of pyrazole moiety, additionally, many anticancer drugs containing pyrazolone, pyrazole, and pyrimidinone core have been reported, such as Metamizole^23^, Propyphenazone^24^, Afloqualone^25^, Betazoleas^26^, Lonazolac^27^.

Considering these, we attempted to create a novel class of new pyrazolone derivatives and investigated their antioxidant, and anticancer impact by elucidating their inhibitory role on YAP/TEAD Hippo signaling pathway (*in-silico* and *in-vitro*). Additionally, their bioavailability and drug-likeness were examined using ADMET.

## Experimental

### Materials and instrumentation

The chemicals and instrumental data are all contained in the supplementary file (Section S1).

### Synthesis of 3-amino-1-phenyl-1H-pyrazol-5(4H)-one (1)

Compound **1** was prepared as previously described by Weissberger^28^.

### Synthesis of 4-((4-acetylphenyl)diazenyl)-3-amino-1-phenyl-1H-pyrazole-5(4H)-one (2)

To a solution of *p*-amino acetophenone (13.7 mmol) in concentrated HCl, a solution of sodium nitrite (12.7 mmol) was added slowly. The freshly prepared *p*-acetyl phenyl diazonium chloride was added to a cooled solution of compound **1** (8.5 mmol) dissolved in pyridine. The reaction mixture was agitated for 2 h, then the product was filtered, and dried.

Orange powder; Yield 74%; mp 91 °C; ^1^H NMR (400 MHz, DMSO-d_6_) δ (ppm): 9.14 (s, 2H, NH_2_), 7.08–8.11 (m, 9H, Ar–H), 2.59 (s, 1H, CH-pyrazolone), 2.20 (s, 3H, CH_3_); IR (KBr) ν: 1600 (CO), 1540 (N=N); Anal. Calcd for C_17_H_15_N_5_O_2_ (321.33): C, 63.54%; H, 4.71%; N, 21.79%. Found: C, 63.34%; H, 4.57%; N, 21.67%.

### Synthesis of 5-amino-4-((Z)-(4-((E)-3-(4-(dimethylamino) phenyl) acryloyl) phenyl) diazenyl)-2-phenyl-2,4-dihydro-3H-pyrazole-3-one (3)

A mixture of ethanolic solution of compound **2** (10.0 mmol), 4-(dimethylamino) benzaldehyde (10.0 mmol), and sodium hydroxide (20.0 mmol) was agitated for 10 h at room temperature, then put into freezing water, filtered, and dried.

Dark brown powder; Yield 81%; mp 114 °C; ^1^H NMR (400 MHz, DMSO-d_6_) δ (ppm): 9.67 (s, 2H, NH_2_), 7.09–8.35 (m, 13H, Ar–H), 7.55 (d, J=11.55 Hz, 1H,=CH-Ar), 6.74 (d, J=12.00 Hz, 1H, COCH=), 3.04 (s, 6H, N(CH_3_)_2_), 2.84 (s, 1H, CH-pyrazolone); ^13^C NMR (101 MHz, DMSO-d_6_) δ (ppm):154.73 (C=O chalcone), 152.51 (C=O pyrazolone), 145.51 (C = N pyrazolone), 111.52–137.28 (Ar–C), 122.98, 138.22 (C-Olefinic), 79.78 (CH pyrazolone), 55.34 (N(CH_3_)_2_); IR (KBr) ν: 3400 (NH_2_), 1660 (C=O), 1520 (C=N),1500 (N=N); MS m/z (%): 452.35 [M^+^];Anal. Calcd for C_26_H_24_N_6_O_2_ (452.20): C,69.01%; H,5.35%; N,18.57%. Found: C,68.91%; H,5.55%; N,18.39%.

### Synthesis of 3-amino-4-((4-(5-(4-(dimethylamino)phenyl)-1-phenyl-4,5-dihydro-1H-pyrazole-3-yl)phenyl) diazenyl)-1-phenyl-1H-pyrazole-5(4H)-one (4)

A mixture of compound **3** (10.0 mmol), and phenylhydrazine (10.0 mmol) was refluxed in acetic acid for 9 h. the mixture was put into freezing water, filtered, and dried.

Green powder; Yield 73%; mp 96 °C; ^1^H NMR (400 MHz, DMSO-d_6_) δ (ppm): 9.57 (s, 2H, NH_2_), 6.68–9.72 (m, 13H, Ar–H), 3.74 (t, J = 10.52 Hz, 1H, CH-CH_2_), 2.91 (s, 6H, N(CH_3_)_2_), 2.67 (s, 1H, CH-pyrazolone), 2.03(d, J = 12.23 Hz, 2H, CH_2_) ; IR (KBr) ν: 1670 (C=O), 1650 (C=N), 1510 (N=N); Anal. Calcd for C_32_H_30_N_8_O (542.25):C,70.83%; H,5.57%; N,20.65%. Found: C,70.75%; H,5.49%; N,20.59%.

### 4-((4-(1-acetyl-5-(4-(dimethylamino)phenyl)-4,5-dihydro-1H-pyrazole-3-yl)phenyl) diazenyl)-3-amino-1-phenyl-1H-pyrazole-5(4H)-one (5)

A mixture of compound **3** (10.0 mmol), and hydrazine hydrate (10.0 mmol) was refluxed in ethanol for 7 h. the mixture was put into freezing water, filtered, and dried.

Dark brown powder; Yield 87%; mp 133 °C; ^1^H NMR (400 MHz, DMSO-d_6_) δ (ppm): 9.67 (s, 2H, NH_2_), 8.49 (s,1H, NH ), 6.69–8.03 (m, 13H, Ar–H), 4.72 (t, J = 11.18 Hz, 1H, CH), 2.99 (s, 1H, CH-pyrazolone), 2.86 (s, 6H, N(CH_3_)_2_), 2.02 (d, J = 11.95 Hz, 2H,CH_2_); IR (KBr) ν: 3150 (NH), 1610 (C = O), 1580 (C = N), 1540 (N = N); MS m/z (%): 466.20 [M^+^]; Anal. Calcd for C_26_H_26_N_8_O (466.22):C,66.94%; H,5.62%; N,24.02%. Found: C,66.86%; H,5.56%; N,23.94%.

### 4-((4-(1-acetyl-5-(4-(dimethylamino)phenyl)-4,5-dihydro-1H-pyrazole-3-yl)phenyl) diazenyl)-3-amino-1-phenyl-1H-pyrazole-5(4H)-one (6)

A mixture of compound **3** (10.0 mmol), and hydrazine hydrate (10.0 mmol) was refluxed in acetic acid for 8 h. The mixture was put into freezing water, filtered, and dried.

Brown powder; Yield 75%; mp 74 °C; ^1^H NMR (400 MHz, DMSO-d_6_) δ (ppm): 9.67 (s, 2H, NH_2_), 6.65–8.39 (m, 13H, Ar–H), 4.67 (t, J = 12.04 Hz, 1H, CH), 2.99 (s, 1H, CH-pyrazolone), 2.84 (s, 6H, N(CH_3_)_2_), 2.27 (d, J = 11.75 Hz, 2H, CH_2_), 1.91 (s, 3H, CH3); ^13^C NMR (101 MHz, DMSO-d_6_) δ (ppm):171.68 (C=O pyrazolone), 166.97 (C=O pyrazole), 159.68 (C=O acetyl), 151.74 (C=N pyrazole), 149.79 (C=N pyrazolone), 111.05–130.42 (Ar–C), 75.22 (CH pyrazolone), 35.81 (N(CH_3_)_2_), 21.24 (CH3) ; IR (KBr) ν: 1650 (C=O), 1540 (C=N), 1500 (N=N); Anal. Calcd for C_28_H_28_N_8_O_2_ (508.23):C,66.13%; H,5.55%; N,22.03%. Found: C,66.11%; H,5.47%; N,21.97%.

### Synthesis of 3-amino-4-((4-(6-(4-(dimethylamino)phenyl)-2-oxo-1,2-dihydropyridine-4-yl)phenyl)diazenyl)-1-phenyl-1H-pyrazole-5(4H)-one (7)

An ethanolic solution of compound **3** (10.0 mmol), and urea (10.0 mmol) was refluxed in HCl for 7 h. the mixture was put into freezing water, filtered, and dried.

Dark brown powder; Yield 74%; mp 145 °C; ^1^H NMR (400 MHz, DMSO-d_6_) δ (ppm): 9.67 (s, 2H, NH_2_), 9.15 (s,1H, NH pyrimidinone), 6.97–8.19 (m, 13H, Ar–H), 5.72 (s, 1H, CH-pyrimidinone), 3.01 (s, 6H, N(CH_3_)_2_), 2.84 (s, 1H, CH-pyrazolone); ^13^C NMR (101 MHz, DMSO-d_6_) δ (ppm):162.37 (C=O pyrazolone), 160.73 (C=N pyrimidinone), 158.53 (C-NH), 155.37 (C=O pyrimidinone), 151.59 (C=N pyrazolone), 111.54–130.00 (Ar–C), 79.80 (CH pyrazolone), 45.54 (N(CH_3_)_2_); IR (KBr) ν: 3400 (NH), 1615 (C=O), 1530 (C=N), 1500 (N=N); Anal. Calcd for C_27_H_24_N_8_O_2_(492.20): C,65.84%; H,4.91%; N,22.75%. Found: C,65.68%; H,4.77%; N,22.67%.

### Synthesis of 3-amino-4-((4-(6-(4-(dimethylamino)phenyl)-2-thioxo-1,2-dihydropyrimidin-4-yl) phenyl) diazenyl)-1-phenyl-1H-pyrazol-5(4H)-one (8)

An ethanolic solution of compound **3** (10.0 mmol), and thiourea (10.0 mmol) was refluxed in NaOH for 8 h. The mixture was put into freezing water, filtered, and dried.

Redish brown powder; Yield 73%; mp 103 °C; ^1^H NMR (400 MHz, DMSO-d_6_) δ (ppm): 9.67 (s, 2H, NH_2_), 9.19 (s,1H, NH pyrimidinthione), 6.75–8.19 (m, 13H, Ar–H), 5.90 (s, 1H, CH-pyrimidinone), 3.13 (s, 6H, N(CH_3_)_2_), 2.61 (s, 1H, CH-pyrazolone); IR (KBr) ν: 3420 (NH), 1618 (C=O), 1540 (C=N), 1513 (N=N); Anal. Calcd for C_27_H_24_N_8_OS (508.18):C,63.76%; H,4.76%; N,22.03%; S,6.30%. Found: C,63.68%; H,4.66%; N,22.01%; S,6.22%.

### Synthesis of (E)-2-amino-6-(4-((3-amino-5-oxo-1-phenyl-4,5-dihydro-1H-pyrazole-4-yl) diazenyl)phenyl)-4-(4-(dimethylamino)phenyl)-4H-pyran-3-carbonitrile (9)

An ethanolic solution of compound **3** (10.0 mmol), and malononitrile (10.0 mmol) was refluxed in HCl for 8 h. The mixture was put into freezing water, filtered, and dried.

Orange powder; Yield 79%; mp 139 °C; ^1^H NMR (400 MHz, DMSO-d_6_) δ (ppm): 9.65 (s, 2H, NH_2_), 9.37 (s, 2H, NH_2_-pyran), 6.59–8.28 (m, 13H, Ar–H), 5.81 (d, J = 9.65 Hz, 1H,=CH Pyran), 5.33(d, J = 11.75 Hz, 1H, CH Pyran), 2.98 (s, 6H, N(CH_3_)_2_), 2.68 (s, 1H, CH-pyrazolone); ^13^C NMR (101 MHz, DMSO-d_6_) δ (ppm):161.10 (C=O pyrazolone), 158.53 (C-NH_2_ pyran), 151.59 (C=N pyrazolone), 123.34 (CN), 111.90–130.27 (Ar–C), 75.08 (CH pyrazolone), 44.61 (C–CN), 23.29 (N(CH_3_)_2_), 22.64 (CH-pyran); IR (KBr) ν: 3430 (NH_2_), 1610 (C=O), 1550 (N=N); Anal. Calcd for C_29_H_26_N_8_O_2_ (518.22):C,67.17%; H,5.05%; N,21.61%. Found: C,67.05%; H,4.95%; N,21.55%.

### Synthesis of (E)-2-amino-6-(4-((3-amino-5-oxo-1-phenyl-4,5-dihydro-1H-pyrazole-4-yl)diazenyl)phenyl)-4-(4-(dimethylamino)phenyl)nicotinonitrile (10)

An ethanolic solution of compound **3** (10.0 mmol), malononitrile (10.0 mmol), and ammonium acetate (20.0 mmol) was refluxed for 10 h. The mixture was put into freezing water, filtered, and dried.

Brown powder; Yield 82%; mp 116 °C; ^1^H NMR (400 MHz, DMSO-d_6_) δ (ppm): 9.45 (s, 2H, NH_2_), 9.15 (s, 2H, NH_2_), 6.71–8.04 (m, 14H, Ar–H), 3.10 (s, 6H, N(CH_3_)_2_), 2.99 (s, 1H, CH-pyrazolone); IR (KBr) ν: 3415 (NH_2_), 1660 (C=O), 1520 (N=N); Anal. Calcd for C_29_H_25_N_9_O (515.22):C,67.56%; H,4.89%; N,24.45%. Found: C,67.48%; H,4.81%; N,24.39%.

### (E)-6-(4-((3-amino-5-oxo-1-phenyl-4,5-dihydro-1H-pyrazol-4-yl)diazenyl) phenyl)-4-(4-(dimethylamino)phenyl)-2-oxo-1,2-dihydropyridine-3-carbonitrile (11)

An ethanolic solution of compound **3** (10.0 mmol), ethyl cyanoacetate (10.0 mmol), and ammonium acetate (20.0 mmol) was refluxed for 9 h. the mixture was put into freezing water, filtered, and dried.

Reddish brown powder; Yield 69%; mp 92 °C; ^1^H NMR (400 MHz, DMSO-d_6_) δ (ppm): 9.78 (s, 2H, NH_2_), 9.27 (s,1H, NH), 6.73–8.18 (m, 14H, Ar–H), 3.00 (s, 6H, N(CH_3_)_2_), 2.71 (s, 1H, CH-pyrazolone); IR (KBr) ν: 3390 (NH), 1620 (C=O), 1530 (N=N); Anal. Calcd for C_29_H_24_N_8_O_2_ (516.20):C,67.43%; H,4.68%; N,21.69%. Found: C,67.35%; H,4.62%; N,21.61%.

### Biological investigations

#### Antioxidant activity using DPPH

The synthesized pyrazolone derivatives’ capacity to scavenge free radicals DPPH was assessed using a modified Zheleva-Dimitrova technique^29^. Also, the DPPH radical was investigated to L-ascorbic acid with concentrations (10–100 μM) that serving as the standard positive control and the scavenging percent was calculated by Eq.  1.

1 $${\text{DPPH radical scavenging activity \% }} = \frac{{{\text{A control}}{-}{\text{ A sample}}}}{{\text{A control}}} \times 100$$

### Docking and ADMET *in-silico* studies

The pyrazolone derivatives’ binding mechanisms to the target protein YAP/TEAD were assessed using molecular docking study. The RCSB protein data (PDB: #3KYS) (https://www.rcsb.org/structure/3KYS) gave the crystal structures of the target^30^. The efficiency of the target protein was increased by removing heteroatoms and water molecules. Additionally, the 2D structures of the generated analogs were created in the cdx format and subsequently transformed into motif files (3D structures) through the utilization of ChemDraw Ultra 8.0 (https://en.freedownloadmanager.org/users-choice/Chemdraw_Ultra_8.0.html). Molegro Virtual Docker (2008) (http://molexus.io/molegro-virtual-docker/) was used to study the enzyme-ligand interaction^31^. The intermolecular interactions between the YAP/TEAD protein and synthetic pyrazolone derivatives were observed using the Discovery Studio 3.5 software (https://discover.3ds.com/discovery-studio-visualizer-download). The online tool SwissADME (http://www.swissadme.ch/) was utilized to estimate ADMET features.

### Anticancer assessments *(in-vitro)*

#### Cell lines

Mammary gland (MCF-7;# *ATCC* HTB-22), colorectal adenocarcinoma (HCT-116; *# ATCC* CCL-247)), hepatocellular carcinoma (HepG-2; #*ATCC* HB-8065), and human lung fibroblast (WI-38; # *ATCC* CCL-75). The ATCC cell line was obtained via the Holding Company for Biological Products and Vaccines (VACSERA), Cairo, Egypt.

#### MTT assay

The MTT test was utilized to ascertain the pyrazolone derivatives’ inhibitory effects on cell growth using the aforementioned cell lines. This colorimetric assay is based on the transformation of yellow tetrazolium bromide (MTT) by mitochondrial succinate dehydrogenase in living cells into a purple formazan derivative. The RPMI-1640 medium was supplemented with 10% fetal bovine serum to cultivate cell lines. At 37° C in an incubator with 5% CO_2_, 100 units/mL of penicillin and 100 µg/mL of streptomycin were introduced as antibiotics. The cell lines were seeded by 1.0 × 10^4^ cells/well in a 96-well plate and kept at 37 °C for 24 h with 5% CO_2_. Following incubation, the cells were subjected to several concentrations of newly synthesized pyrazolone derivatives and left for 48 h incubation.20 µL of a 5 mg/mL MTT solution was added and incubated for 4 h after the drug treatment. To dissolve the purple formazan produced in each well, 100 µL of dimethyl sulfoxide (DMSO) was applied. The colorimetric test is performed at 630 nm absorbance and recorded using a plate reader (EXL 800, USA)^32^.

### Statistical analysis

GraphPad Prism 6 (San Diego, CA) (GraphPad Prism 6, https://www.graphpad.com/scientific-software/prism/) was used to calculate the IC_50_ values and the data was expressed as mean ± SE.

## Results and discussion

### Chemistry of the novel compounds

**Fig. 2:**
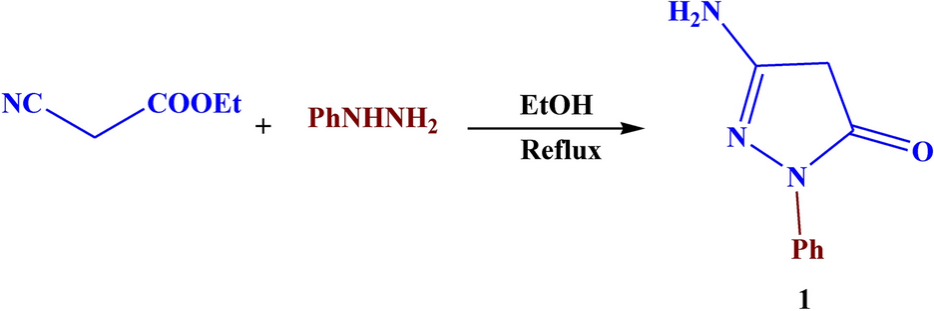
The synthesis route of compound **1.**

Initially, compound **1** was prepared through the reaction of ethyl cyanoacetate and phenyl hydrazine as described previously by Weissberger^28^ as illustrated in Fig. 2.

**Fig. 3:**
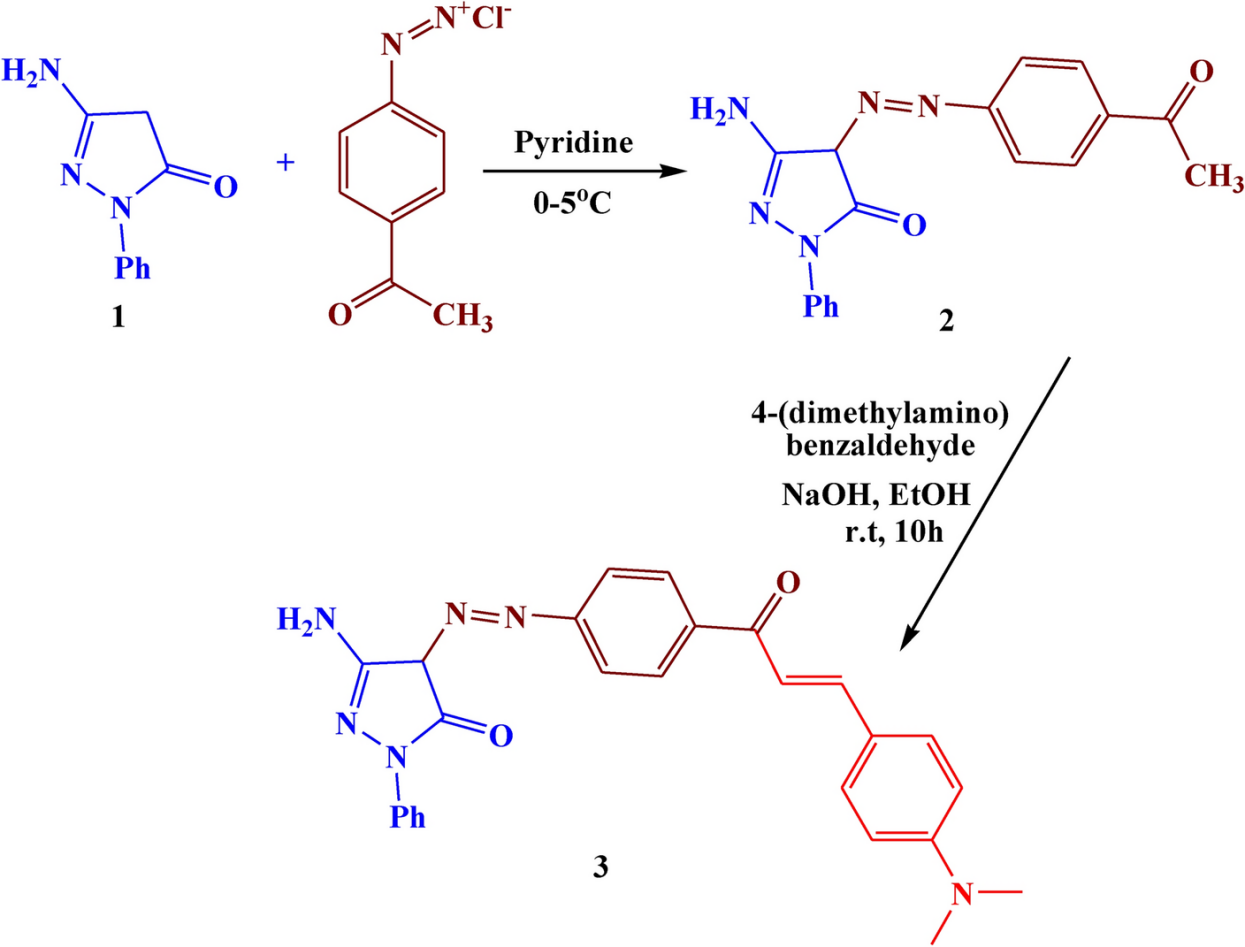
The synthesis route of compound **2, 3**

The coupling of *p*-acetyl phenyl diazonium chloride with compound **1** led to the formation of azo pyrazolone **2** with 74% yield (Fig. 3). The structure of compound **2** was confirmed with elemental analyses and different spectral data. The IR analysis showed absorption bands at 1600 cm^−1^ for the C=O group and 1540 cm^−1^ for the N=N group. The ^1^H NMR spectrum revealed a new singlet signal resonated at *δ* 2.20 ppm attributed to CH_3_ of the acetyl group.

Further modification of compound **2** with 4-(dimethylamino) benzaldehyde in a basic medium led to the formation of pyrazolone chalcone **3** with 81% yield (Fig. 3). The IR analysis showed absorption bands at 1660 cm^−1^ for C=O group, 1520 cm^−1^ for C=N group, and 1500 cm^−1^ for N=N group. The ^1^H NMR spectrum revealed three new signals resonated at *δ* 7.55, 6.74, and 3.04 ppm attributed to two olefinic protons, and N(CH_3_)_2_ respectively.

**Fig. 4:**
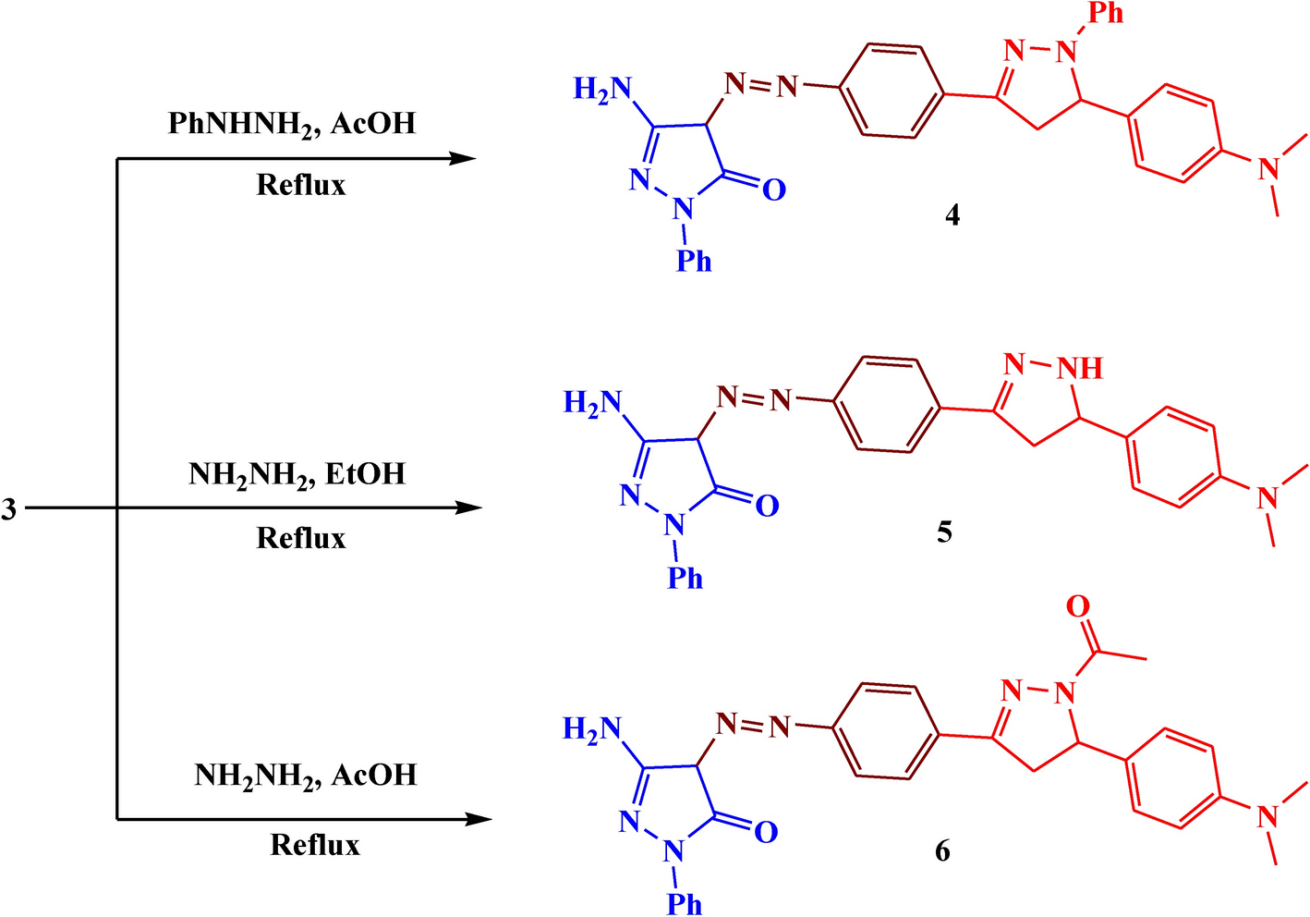
The synthesis route of compounds **4–6.**

The reaction of compound **3** with phenylhydrazine in an acidic medium led to the formation of compound **4** with 73% yield (Fig. 4). The IR showed different bands at 1670, 1650, and 1510 cm^−1^ which belonged to C=O, C=N, and N=N respectively. Its ^1^H NMR spectrum revealed the presence of a new triplet signal at *δ* 3.74 ppm due to CH-pyrazole, and a doublet signal at *δ* 2.03 ppm assigned to CH_2_-pyrazole.

**Fig. 5:**
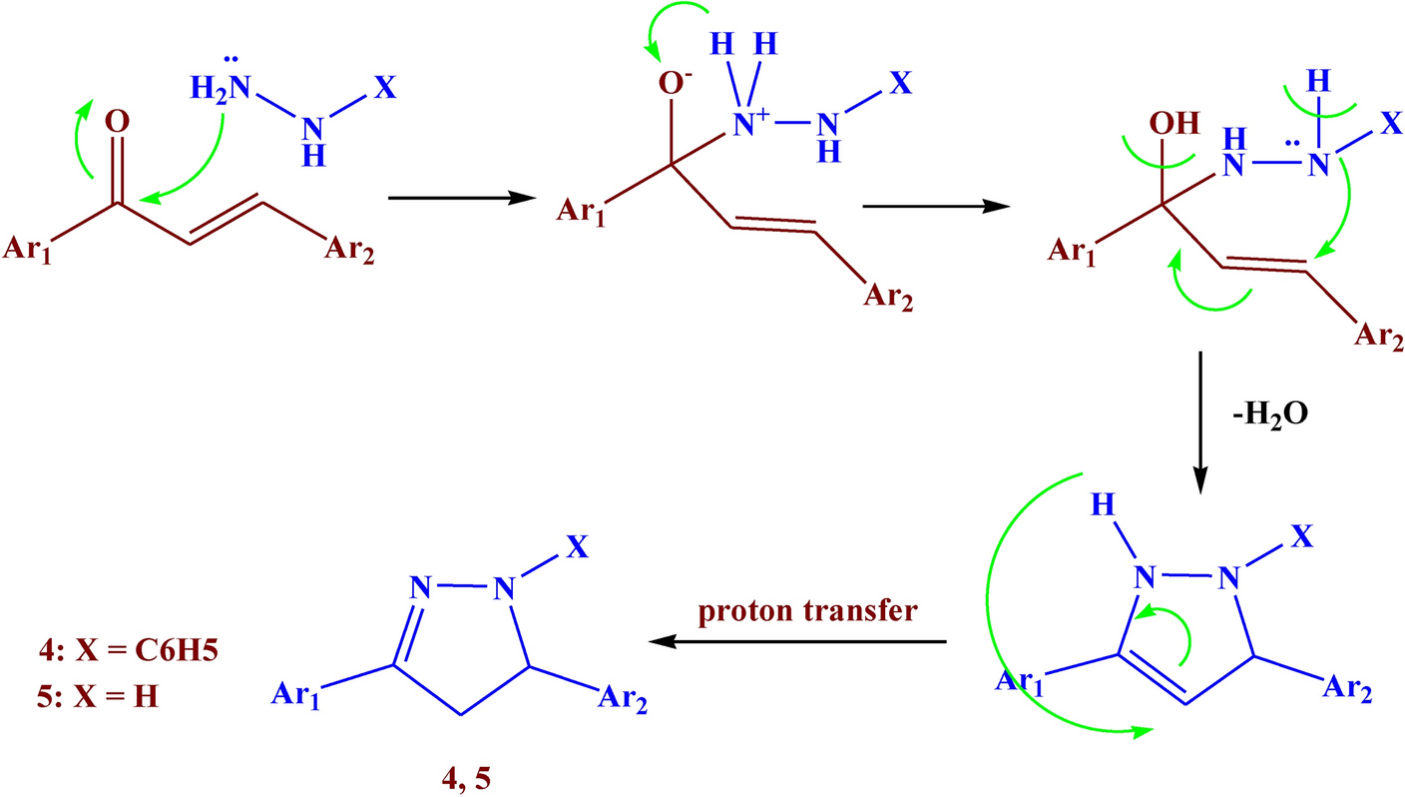
Suggested mechanism for compounds **2, 3**

**Fig. 6:**
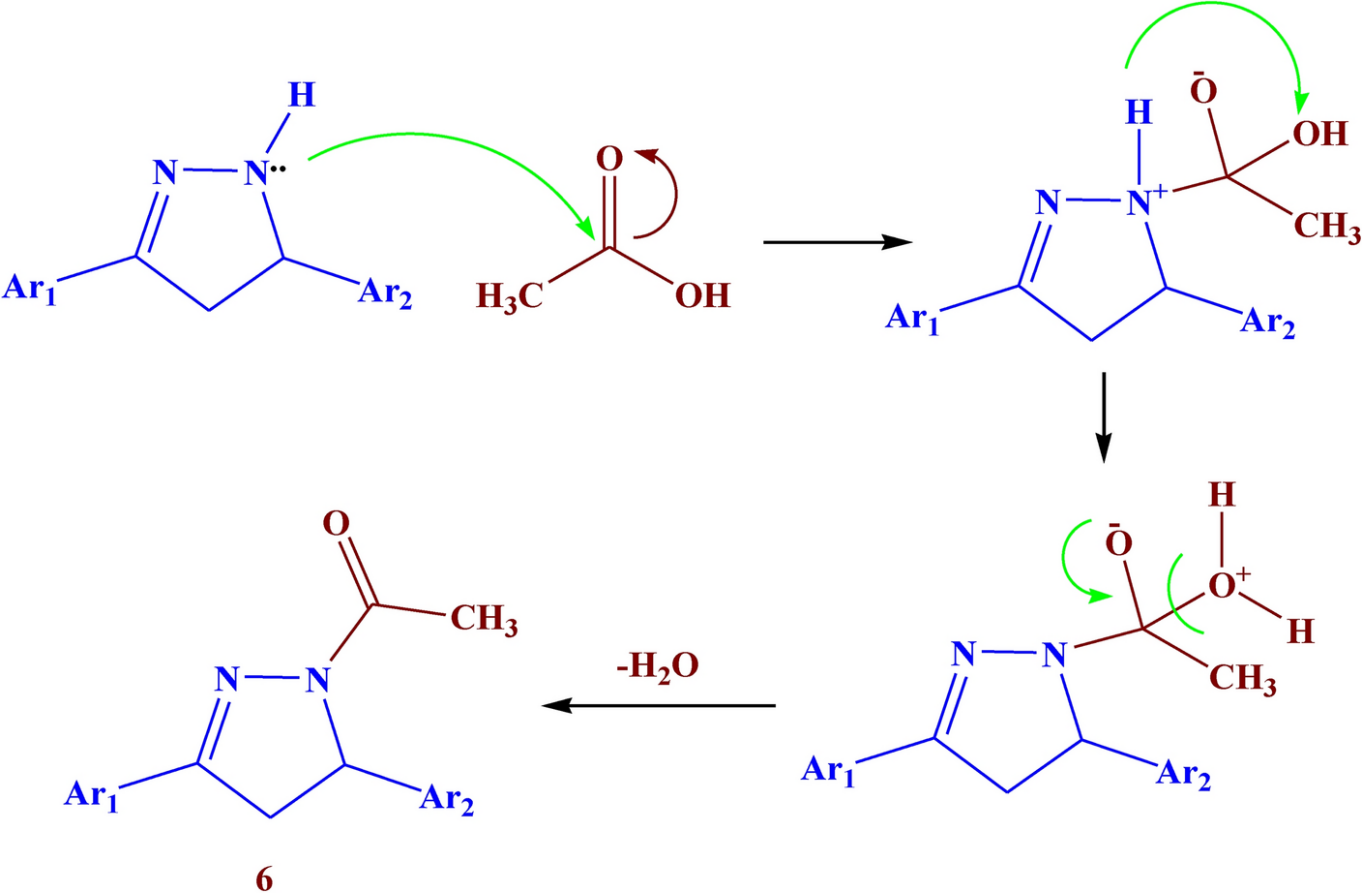
Suggested mechanism for compound **6.**

Additionally, the reaction of the ethanolic solution of compound **3** with hydrazine hydrate led to the formation of compound **5** with 87% yield. The reaction of compound** 3** with hydrazine hydrate in acetic acid glacial led to the formation of compound **6** with 74% yield. (Fig. 4). The IR of compound **5** showed absorption bands at 3150, 1610, 1580, and 1540 cm^−1^ which belonged to NH, C=O, C=N, and N=N respectively. Its ^1^H NMR spectrum showed a new singlet signal at *δ* 8.49 ppm due to NH-pyrazole, a triplet signal at *δ* 4.72 ppm due to CH-pyrazole, and a doublet signal at *δ* 2.02 ppm assigned to CH_2_-pyrazole. The IR of compound **6** showed different bands at 1650, 1540, and 1500 cm^−1^ which belonged to C=O, C=N, and N=N respectively. Its ^1^H NMR spectrum showed a new triplet signal at *δ* 4.76 ppm due to CH-pyrazole, a doublet signal at *δ* 2.27 ppm assigned to CH_2_-pyrazole, and a singlet signal at *δ* 1.91 ppm due to CH_3_ of acetyl group. The suggested mechanism for the synthesis of compounds **4–6** was illustrated in Figs. 5, 6.

**Fig. 7:**
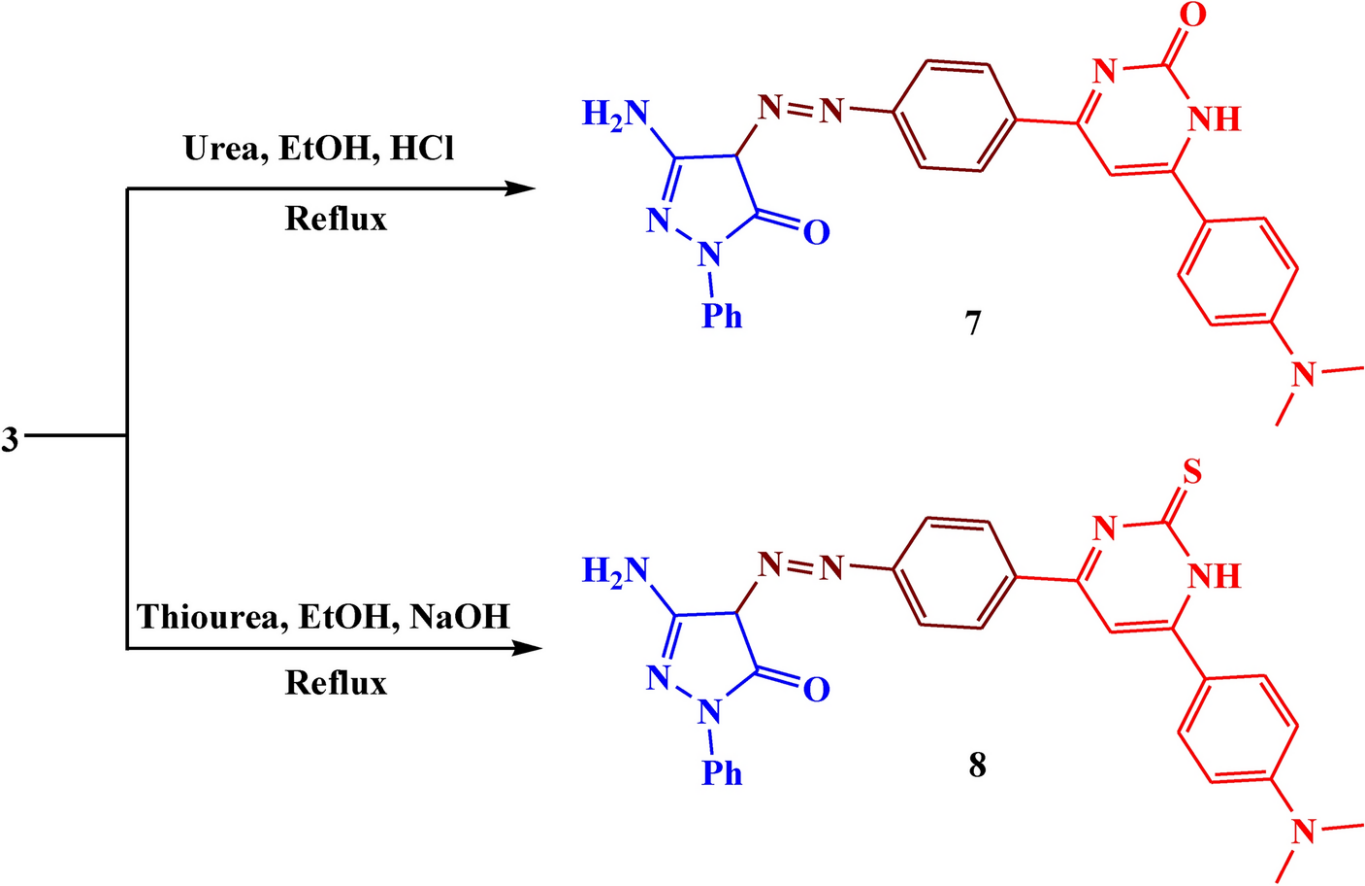
The synthesis route of compounds **7, 8**

**Fig. 8:**
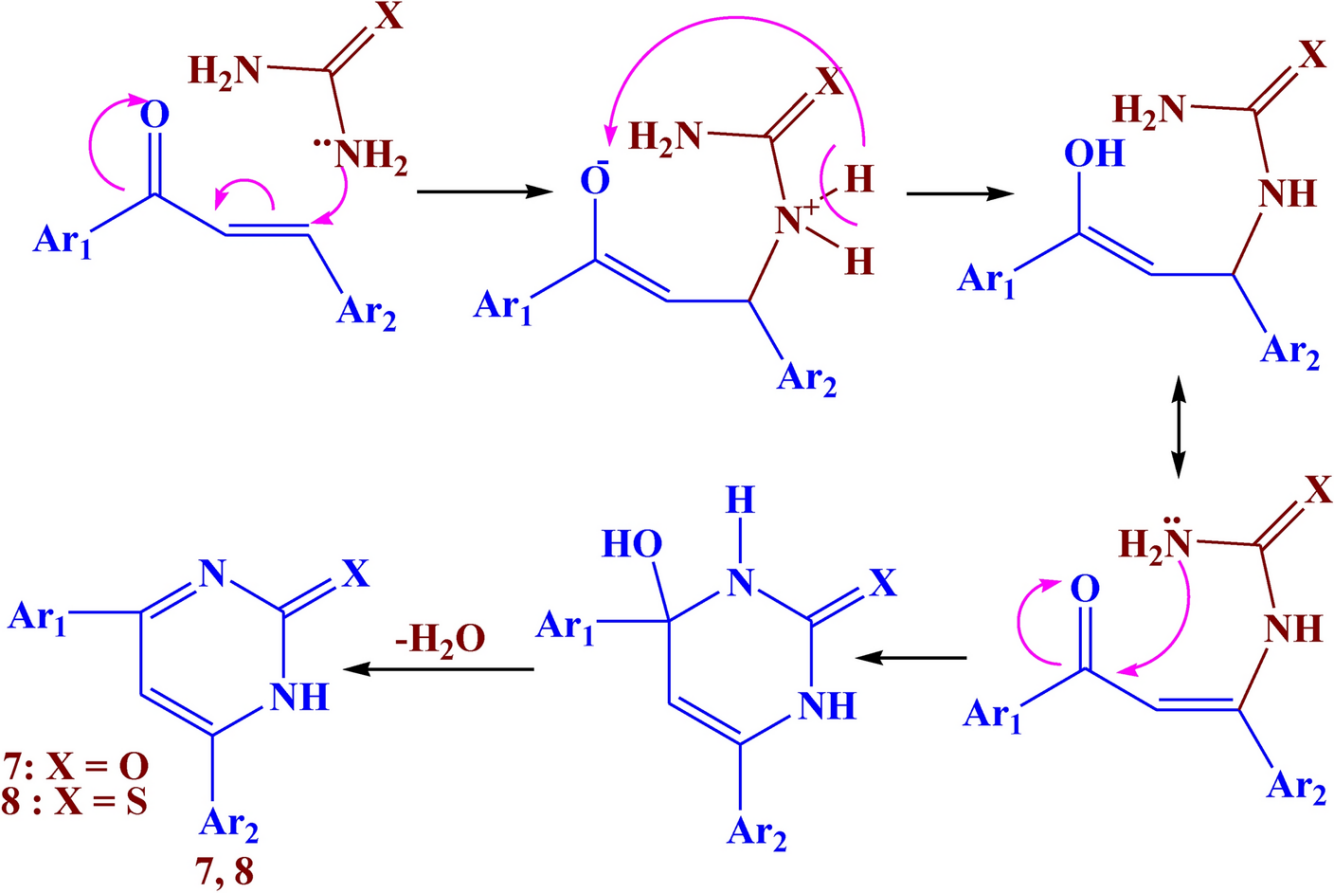
Suggested mechanism for compounds **7, 8**

As shown in Fig. 7, the reaction of compound **3** with urea in an acidic medium and thiourea in a basic medium led to the formation of compounds **7, and** **8**. The IR spectrum of these compounds showed absorption bands at 3400–3420, 1610–1615, 1530–1540, and 1500–1513 cm^−1^ which belonged to NH, C=O, C=N, and N=N respectively. The ^1^H NMR spectrum revealed the presence of a new singlet signal at *δ* 9.15–9.19 characteristic of NH proton, and a singlet signal at *δ* 5.72–5.90 ppm due to CH-pyrimidinone. The suggested mechanism for the synthesis of compounds **7, and** **8** is illustrated in Fig. 8.

**Fig. 9:**
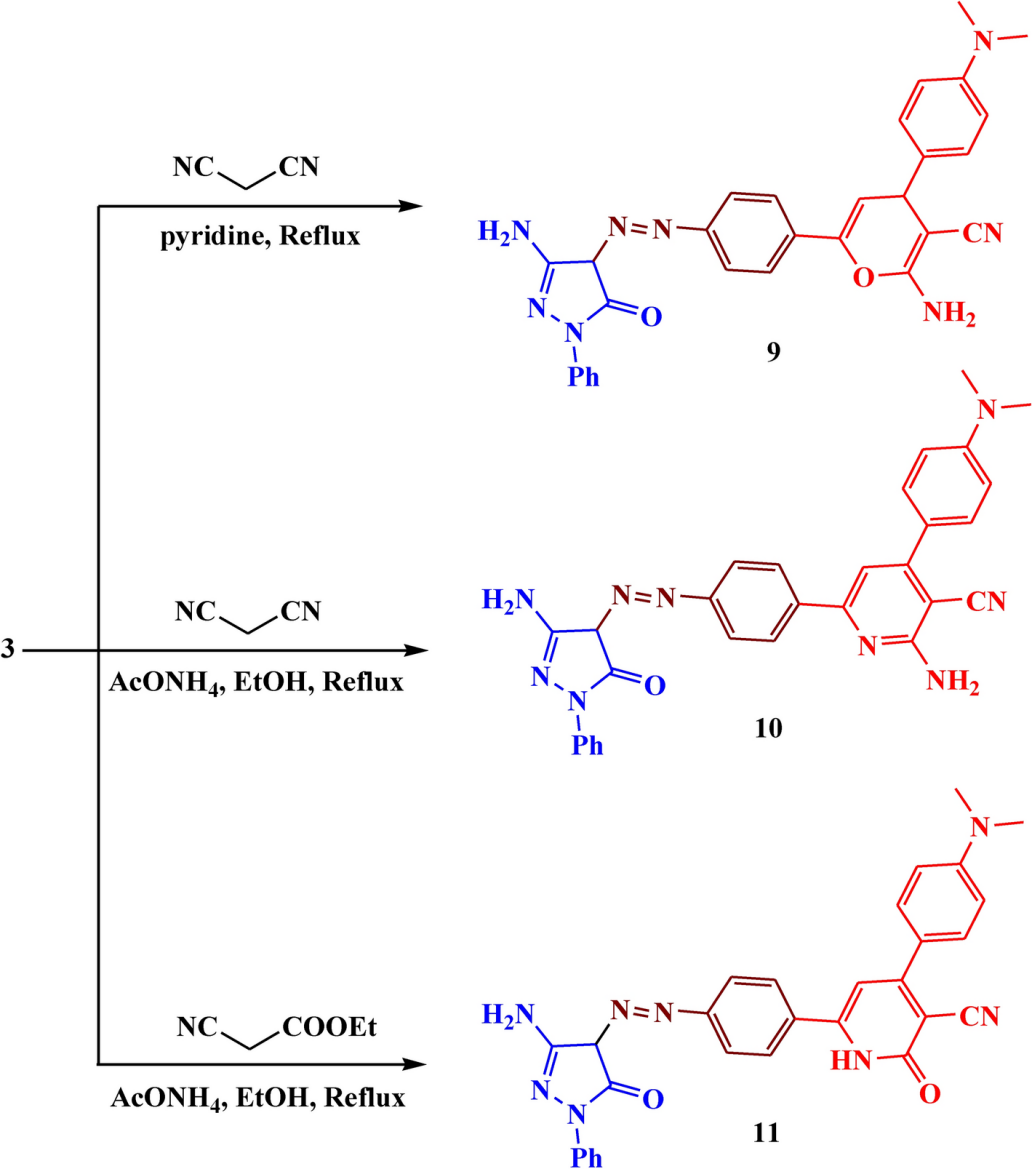
The synthesis route of compounds **9–11.**

Analogously, the reaction of compound** 3** with malononitrile in pyridine led to the formation of compound **9** with a 79% yield. While the reaction with malononitrile in ammonium acetate led to the formation of compound **10** with 82% yield. (Fig. 9). The IR of compound **9** showed different bands at 3430, 1610, and 1550 cm^−1^ which belonged to NH_2_, C=O, and N=N respectively. Its ^1^H NMR spectrum showed a new singlet signal at *δ* 9.37 ppm due to NH_2_ protons, a doublet signal at *δ* 5.81 ppm due to=CH-pyran, and a doublet signal at *δ* 5.33 ppm due to CH-pyran. The IR of compound **10** showed absorption bands at 3415, 1600, and 1520 cm^−1^ which belonged to NH_2_, C=O, and N=N respectively. Its ^1^H NMR spectrum showed a new singlet signal at *δ* 8.49 ppm due to NH_2_ protons.

**Fig. 10:**
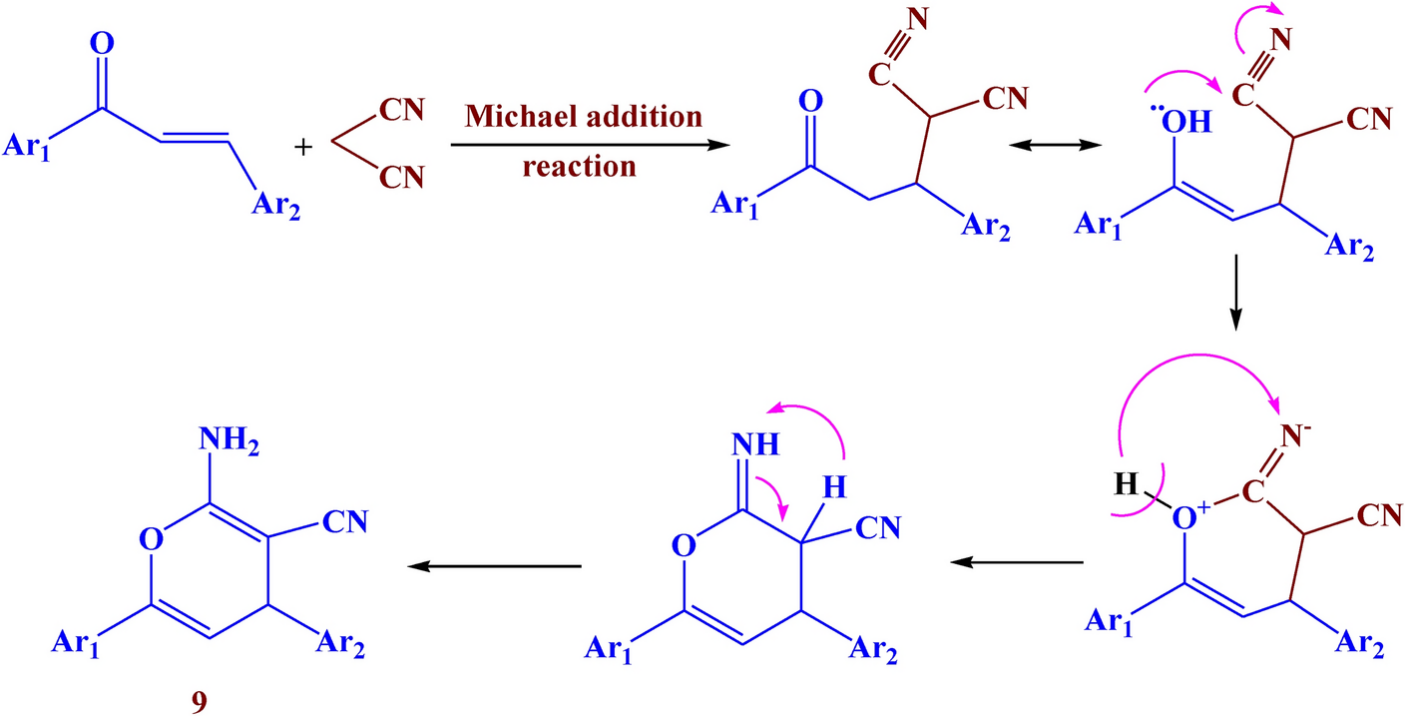
Suggested mechanism for compound **9.**

**Fig. 11:**
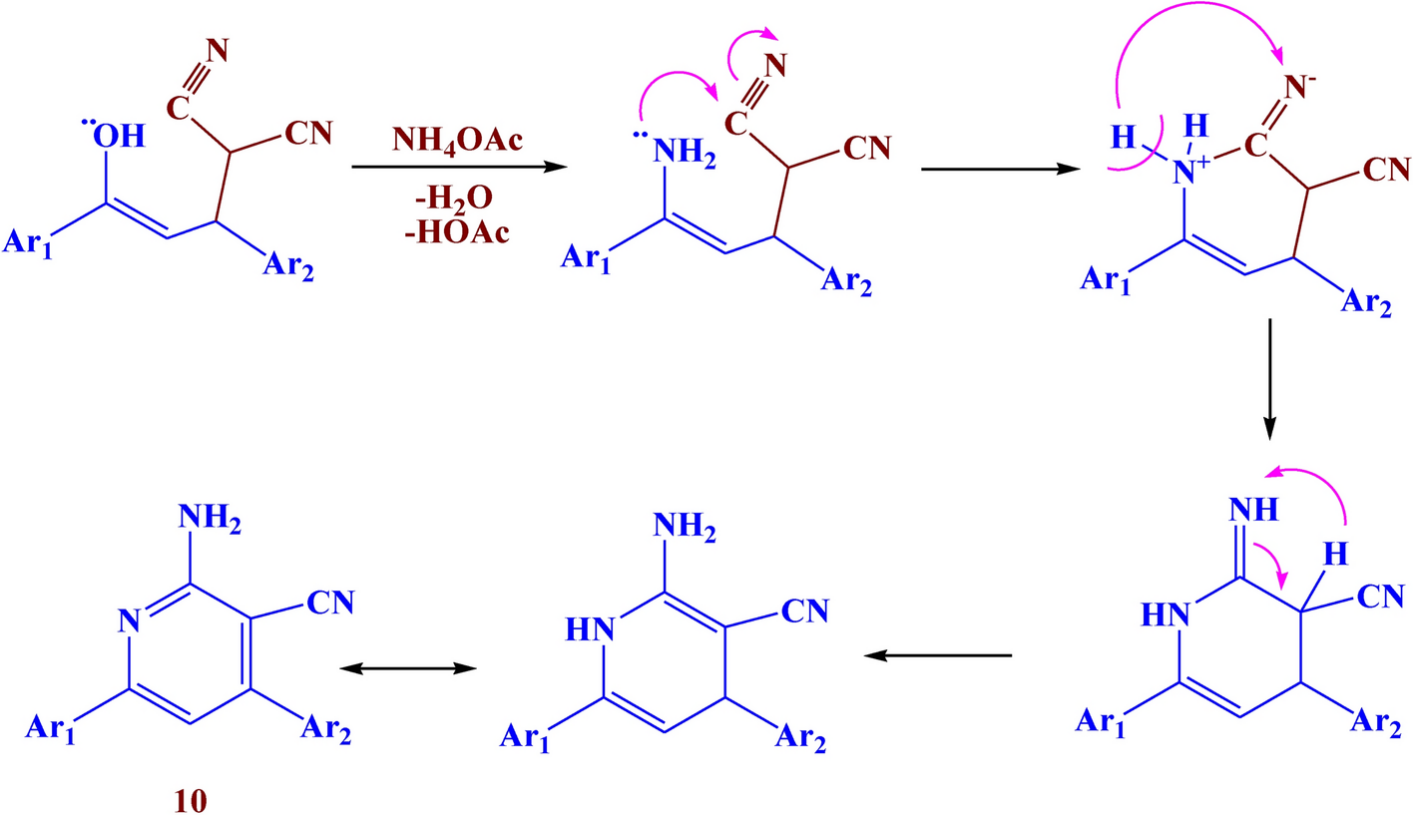
Suggested mechanism for compound **10.**

**Fig. 12:**
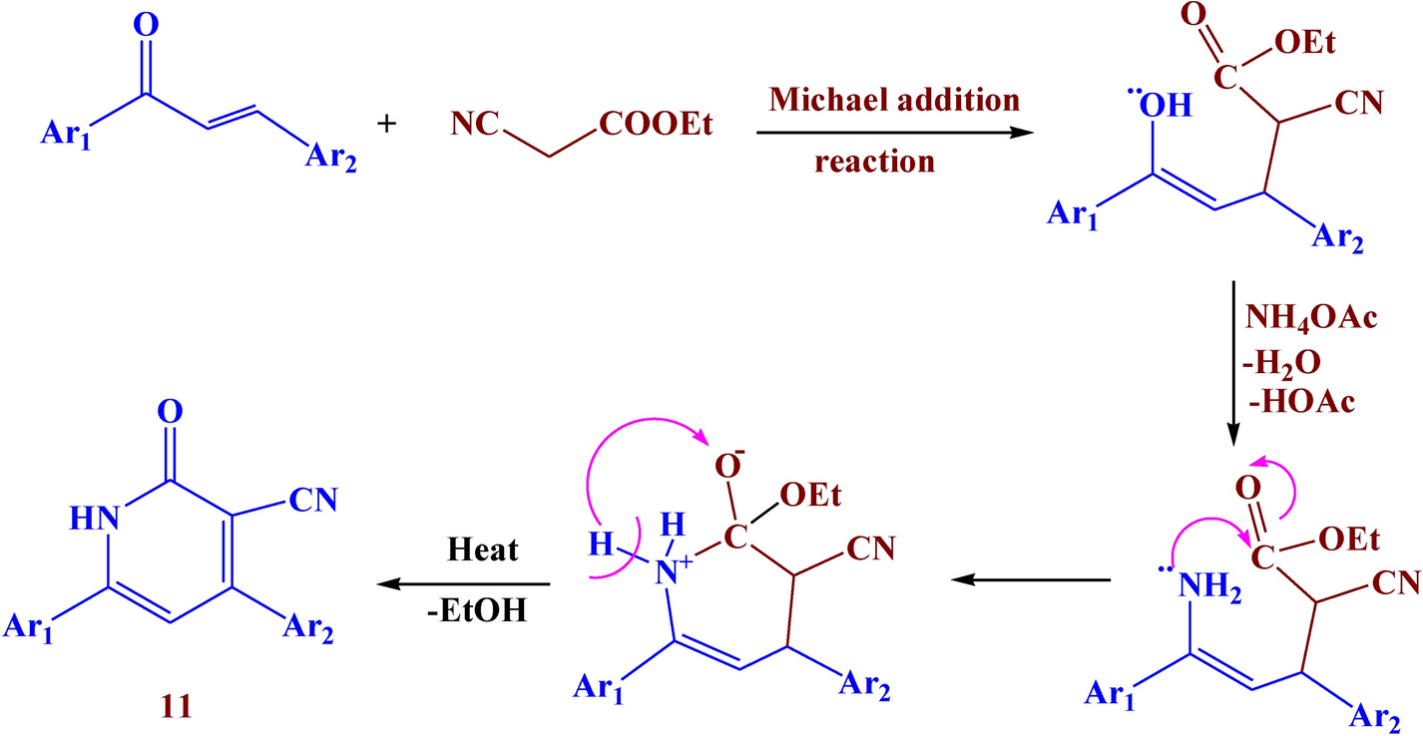
Suggested mechanism for compound **11.**

Finally, the reaction of compound **3** with ethyl cyanoacetate afforded compound **11** with 69% yield. The IR spectra showed different bands at 3390, 1620, and 1530 cm^−1^ which belonged to NH, C=O, and N=N respectively. Its ^1^H NMR spectrum showed a new singlet signal at *δ* 9.27 ppm due to the NH proton. The suggested mechanism for the synthesis of compounds **9–11** was illustrated in Figs. 10, 11, and 12

### Antioxidant capacity

**Fig. 13:**
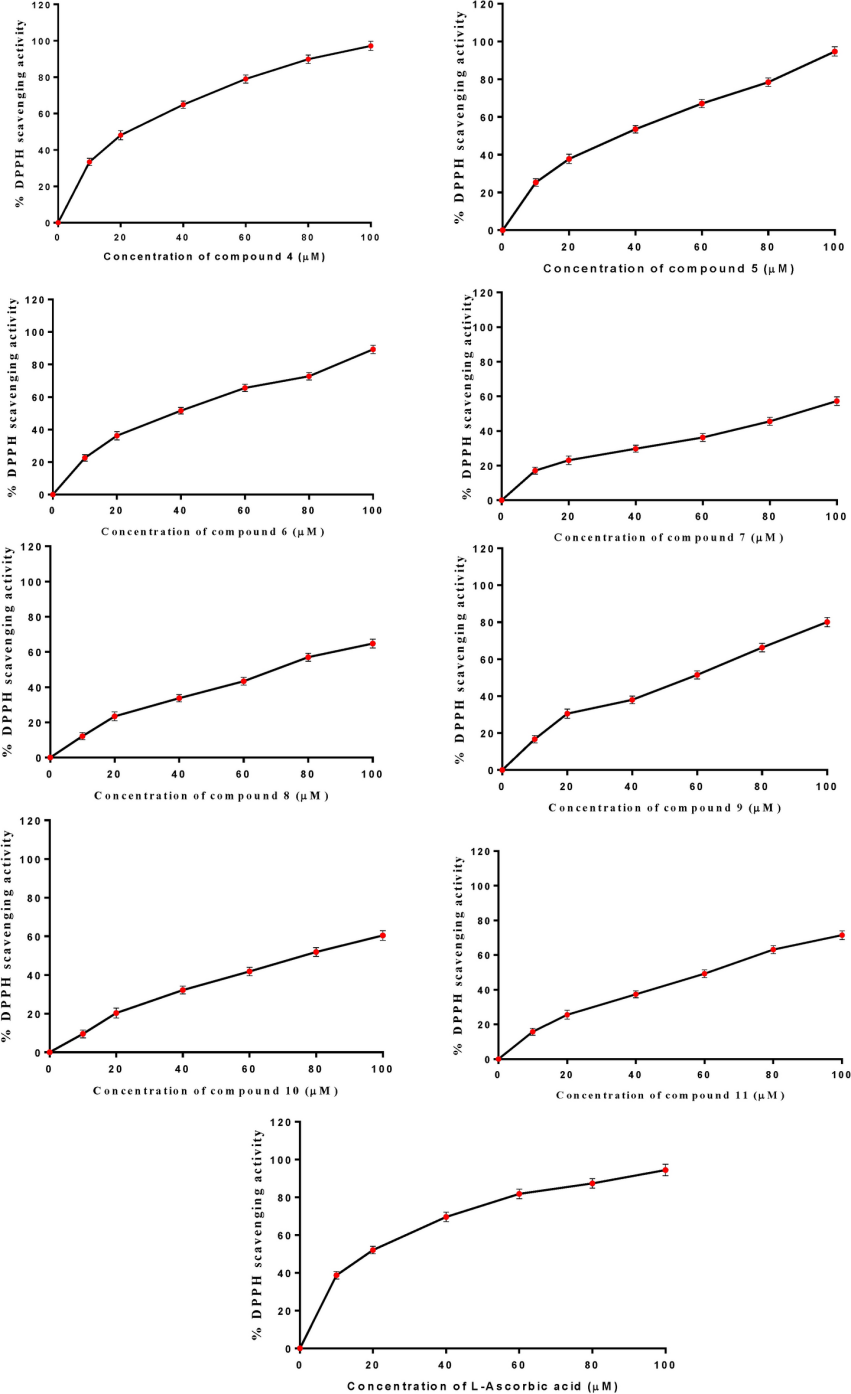
The antioxidant scavenging activity of all novel pyrazolone derivatives (**4–11)** using DPPH.

**Fig. 14:**
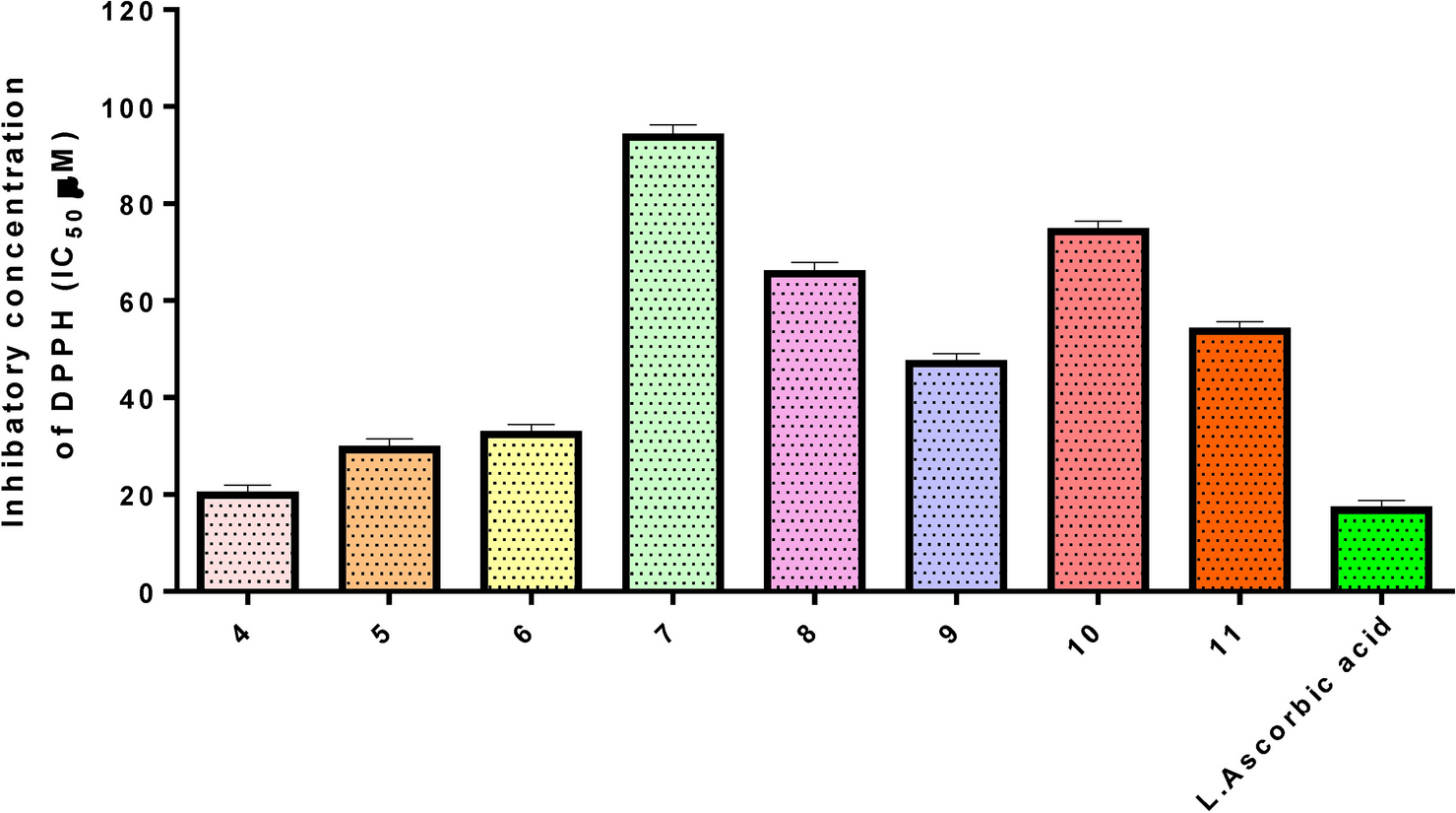
The DPPH inhibitory concentrations (IC_50_) of pyrazolone derivatives (**4–11).**

Using the stable DPPH radical, the antioxidant capacity of these newly synthesized pyrazolones was assessed by monitoring the change in absorbance generated, as illustrated in Figs. 13 and 14. It was elucidated that the antioxidant activity rose along with the concentration of pyrazolone derivatives. Compound **4** showed a higher DPPH scavenging activity with IC_50_ value (19.88 ± 0.15 μM) compared to the standard L-ascorbic acid (IC_50_ = 16.81 ± 0.10 µM). Furthermore, our results observed that compounds **5, 6,** and **9** showed a moderate scavenging impact, with activities equal to 29.28 ± 0.16, 32.40 ± 0.20 and 47.07 ± 0.24 µM respectively. Whereas compounds **7, 8, 10,** and **11** demonstrated a weak capacity to quench free radicals with IC_50_ 93.7 ± 0.47, 65.54 ± 0.32, 74.30 ± 0.38 and 53.67 ± 0.29 µM respectively compared to the standard L-ascorbic acid.

### Docking studies

Molecular docking is a powerful tool for quickly and accurately estimating protein–ligand complex binding energies and biomolecular conformations, it has been widely used to identify novel drugs. The new pyrazolone derivative ligands (**4–11**) in this instance were docked into YAP/TEAD as a well-known and enticing therapeutic target protein-protein complex in the Hippo signaling pathway for the creation of anticancer medications. The Hippo pathway has been the subject of numerous studies in the domains of oncology and regenerative medicine, which have also indicated its possible significance as inhibitory targets for drug development or as regulatory factors in human biology. The Hippo pathway is generally composed of two constitutive modules: the downstream transcriptional modules, which primarily involve the YAP/TEAD complex, and the upstream serine/threonine kinase cascade, which includes MST1/2 and LATS1/2^33^.

**Fig. 15:**
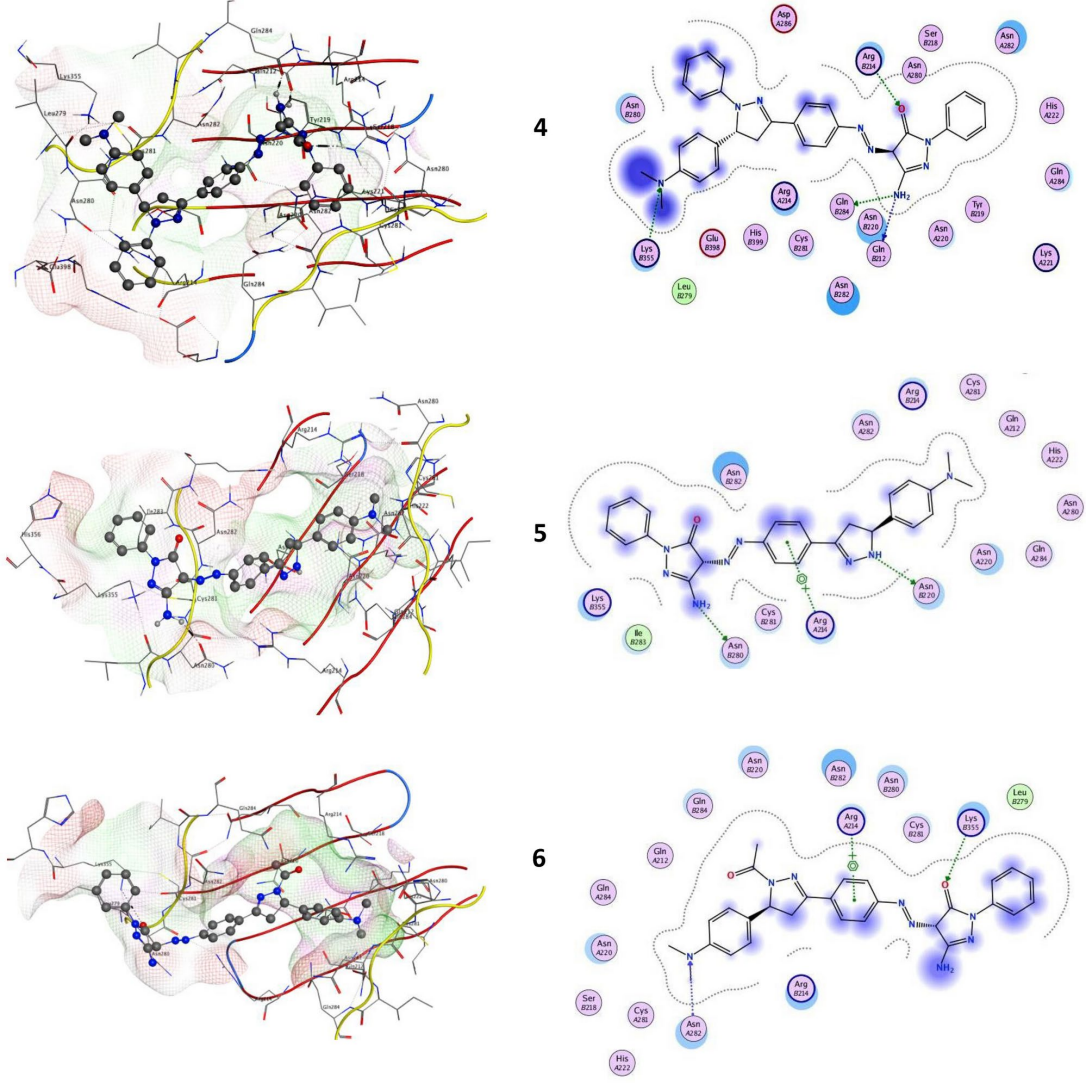

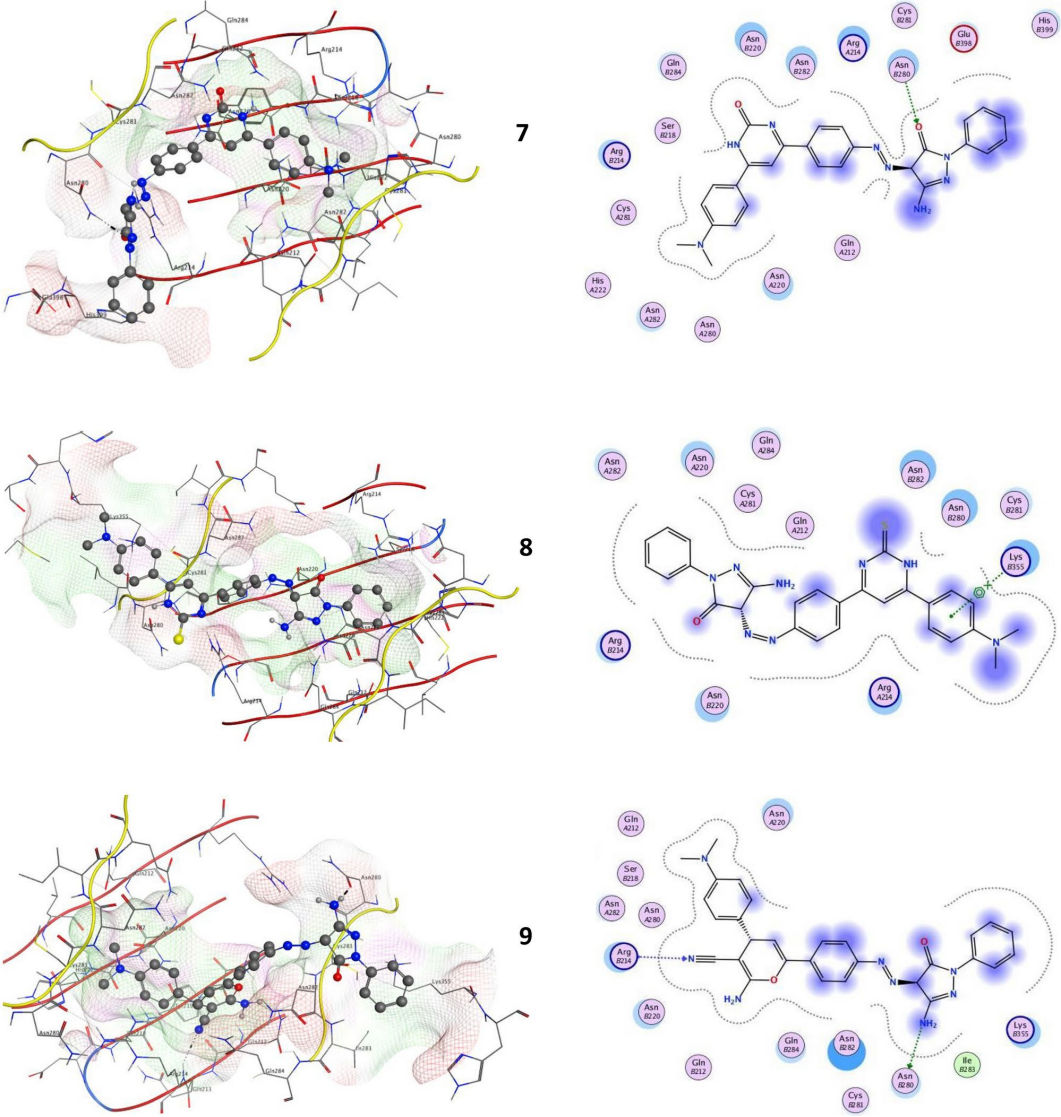

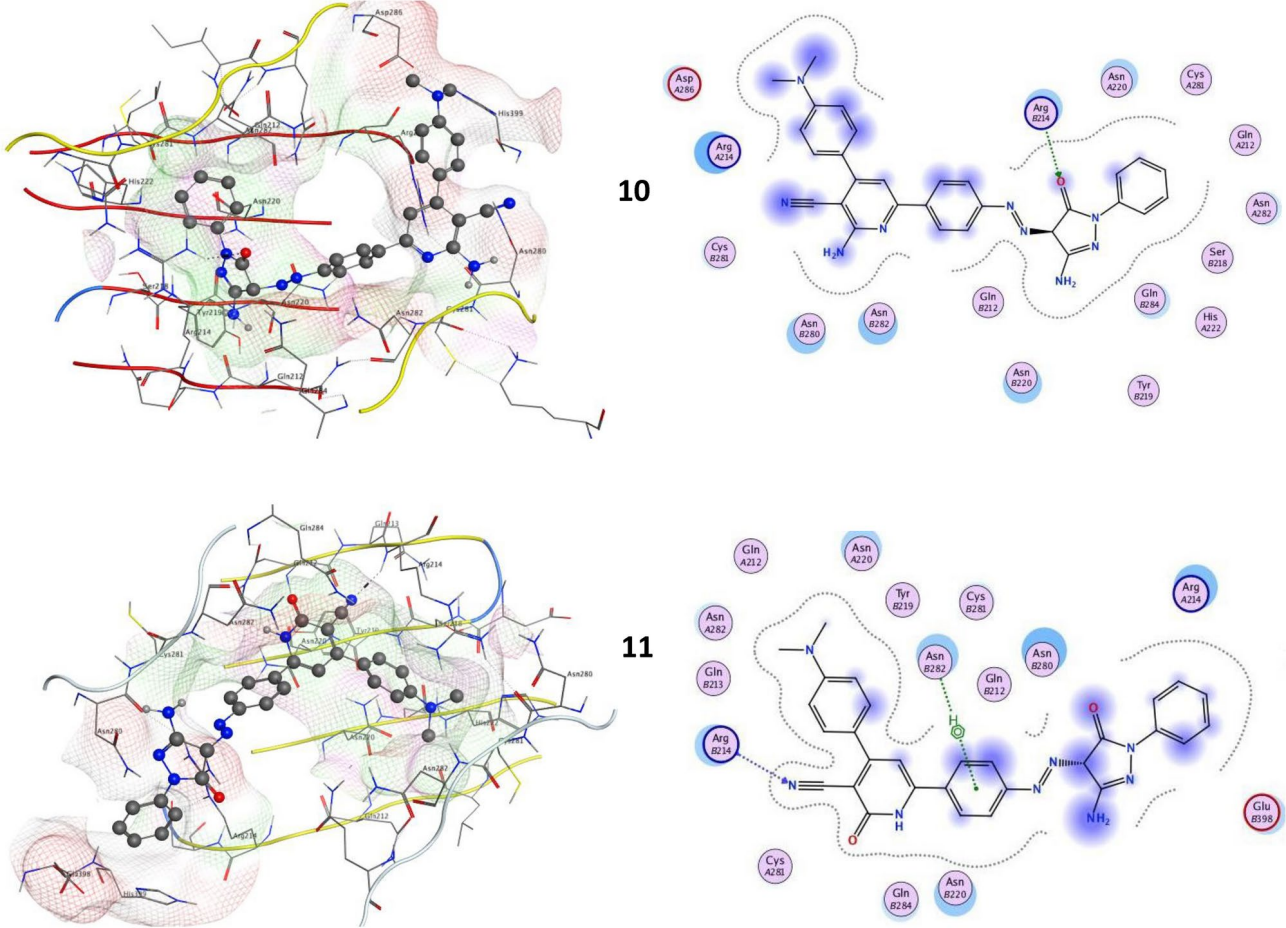
2D and 3D molecular interaction network for all novel pyrazolone derivatives (**4–11**) with the target YAP/TEAD mediated Hippo signaling pathway.

Oncogenes as Yes-associated protein (YAP) was closely homolog with the PDZ-binding motif (TAZ). YAP and TAZ function as transcription co-activators, forming a binding complex with DNA to trigger target gene transcription and consequent transformation activity. The transcriptional enhancer factor (TEA)-domain (TEAD) family is primarily responsible for mediating key transforming activities of YAP and TAZ, such as those related to cell proliferation, invasion, and metastasis. In YAP-dependent cancer models, the YAP/TEAD genetic and pharmacological interactions dramatically reduced tumorigenesis^34^. Thus, the YAP/TEAD complex has emerged as an attractive target for the development of anti-cancer drugs^35^. All novel pyrazolone’s interactions with target YAP/TEAD protein were described in Table 1; Fig. 15. Our results elucidated that compound **4** exhibited the most binding energy against target YAP/TEAD protein with a value equal to − 9.670 kcal/mol. Furthermore, compounds **5, 6,** and **9** elucidated moderate inhibitory effect with binding energies equal to -8.081 and − 7.814 and − 7.113 kcal/mol respectively. On the other hand, compounds **7**, **8**, **10,** and **11** observed slightly weak binding energy equal to − 6.530, − 4.605, − 5.203, and − 7.005 kcal/mol respectively. Therefore, compound **4** was strongly recommended to be used as an anticancer agent via its prospective inhibitory effect on the YAP/TEAD-mediated Hippo signaling pathway.

**Table 1:** Docking scores of all synthesized compounds (**4–11)** with the target protein.

Compounds	YAP/TEAD target Hippo signaling protein	
	Docking score (ΔGbind)	Docked complex (amino acid–ligand) interactions
**4**	− 9.670	H-donor GLNB284 GLNB212 H-acceptor ARGB214 LYSB355 Electrostatic interactions ASNB282 ASNB220 ARGA214 ASNA282 GLNA284 CYSB281
**5**	− 8.081	H-donor ASNB220 ASNB280 π-Hydrogen ARGA214 Electrostatic interactions ARGB214 ASNA220 ASNB282 LYSB355 ASNA282
**6**	− 7.814	H-acceptor LYSB255 ASNA282 π-Hydrogen ARGA214 Electrostatic interactions ASNB280 CYSB281 ASNB282 ASNB220 GLNB284 ARGB214 ASNA220
**7**	− 6.530	H-acceptor ASNB280 Electrostatic interactions ARGA214 ASNB282 ASNB220 ASNA220 ARGB214 GLNB284 HISB298
**8**	− 4.605	π-hydrogen LYSB355 Electrostatic interactions ASNB280 ASNB282 ASNA220 ASNA282 ARGA214 ASNB220 ARGB214
**9**	− 7.113	H-donor ASNB280 H-acceptor ARGB214 Electrostatic interactions LYSB355 ASNB282 GLNB284 ASNB220 ASNA220
10	− 5.203	H-acceptor ARGB214 Electrostatic interactions ASNA220 GLNB284 ASNB220 ASNB282 ASNB280 ARGA214
**11**	− 7.005	H-acceptor ARGB214 π-hydrogen ASNB282 Electrostatic interactions ARGA214 ASNB280 ASNA220 ASNB220 ASNA282 GLUB398

### ADMET features

**Fig. 16:**
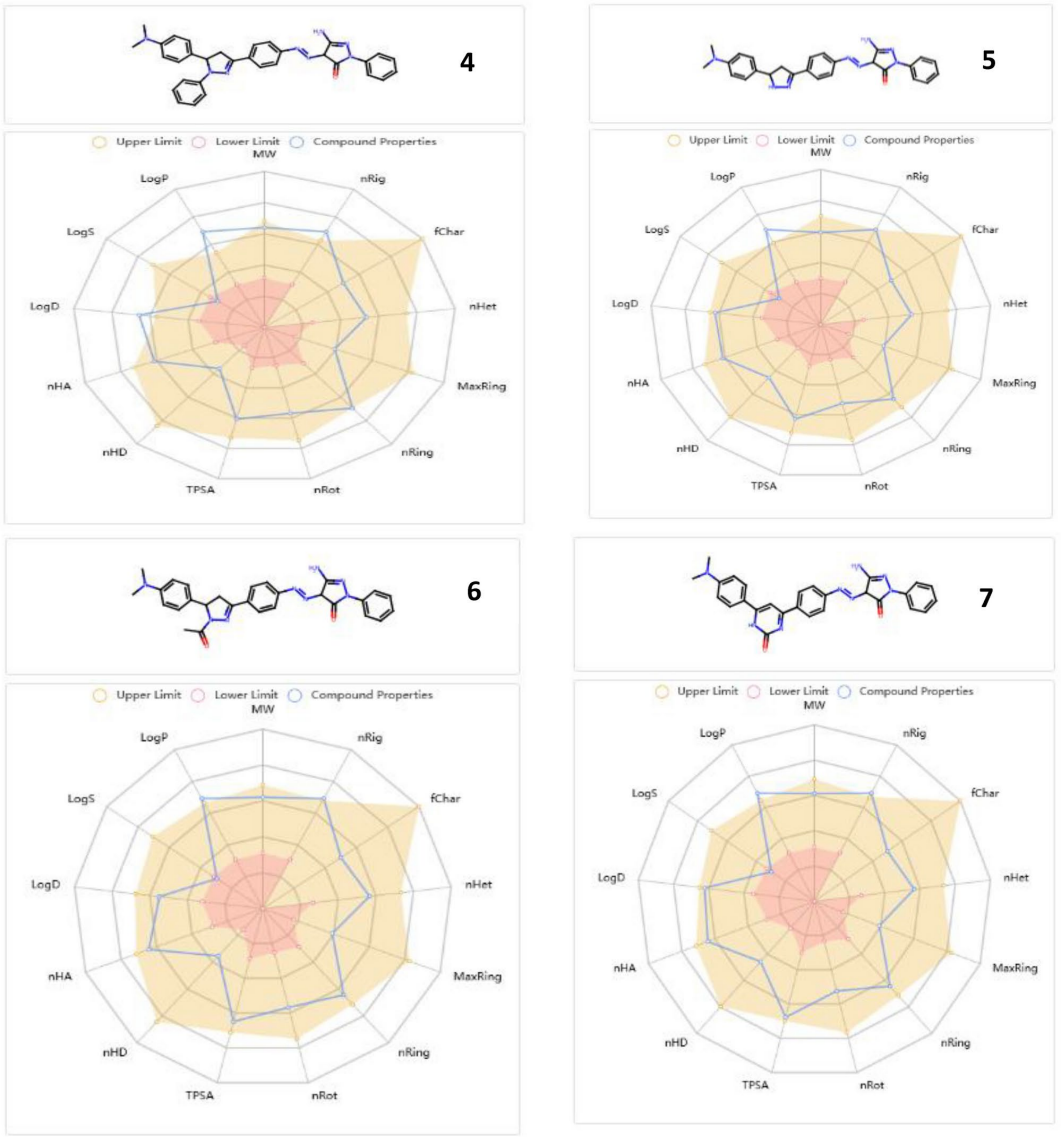

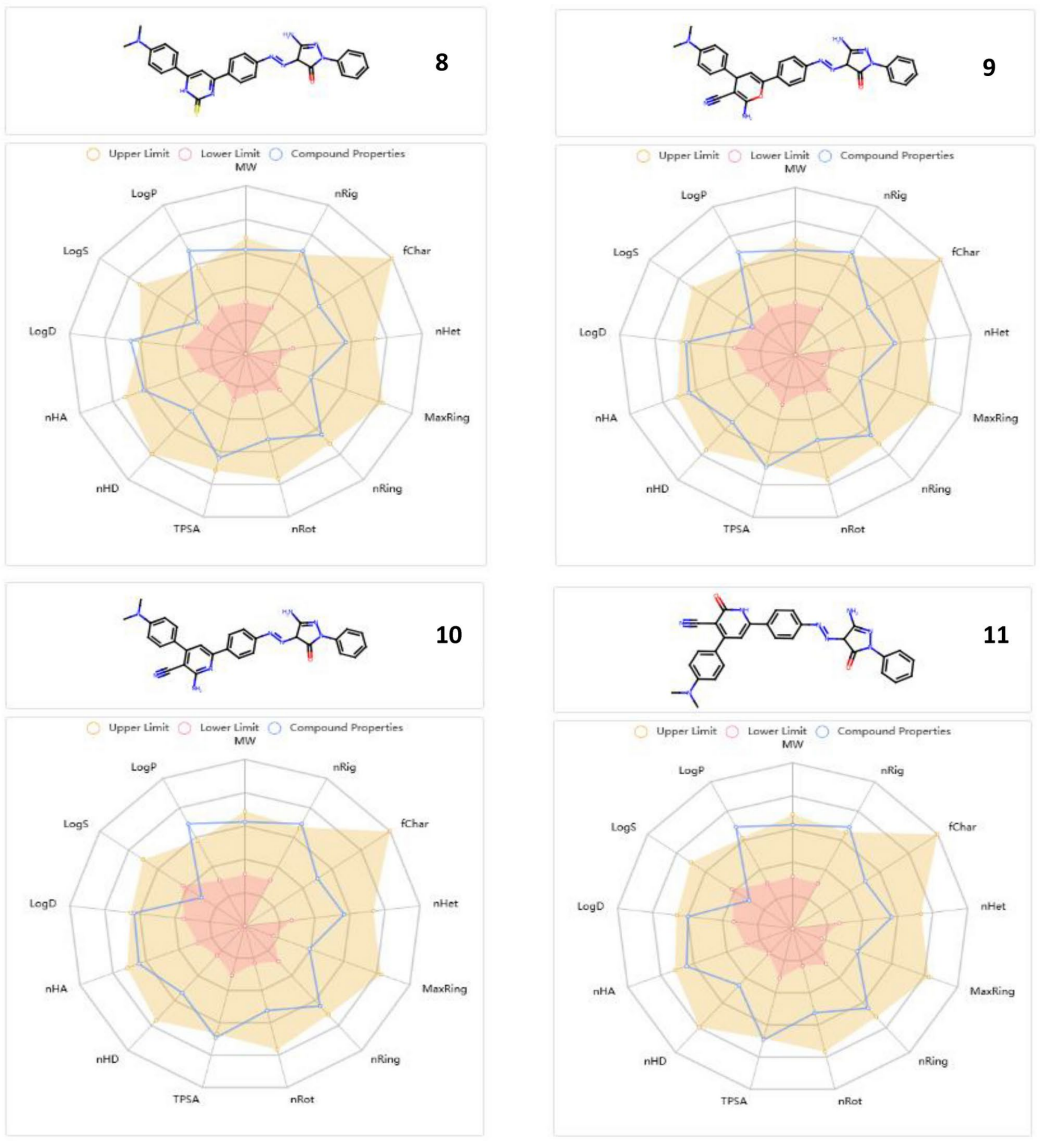
Bioavailability radar plot for all pyrazolone derivatives** (4–11).**

Figure 16 ascertains the bioavailability and drug likeness properties of the examined pyrazolone derivatives (**4–11)**. The total polar surface area (TPSA) of the pyrazolone derivatives (**4–11**) ranged from 102.25 to 149.35 Å2, which means that they had adequate oral bioavailability and satisfied all the criteria for outstanding permeability. They also showed how to demonstrate flexibility by showing rotatable bonds between 0 and 10. Their enhanced solubility in cellular membranes was facilitated by their hydrogen bound accepted (HBA) and hydrogen bound donated (HBD) values being in the fulfilled range. Table 2 illustrates that octanol/water partition coefficient (log p) values less than 5 were indicative of good lipophilicity features. Furthermore, the pyrazolone derivatives had higher human intestinal absorption (% HIA) ratings in accordance with the ADMET criteria, indicating that the human intestinal could absorb them more effectively. The tested pyrazolone derivatives have a great safety profile for the central nervous system (CNS) because they do not cross the blood–brain barrier. Last but not least, every AMES toxicity and carcinogenicity test result was negative, demonstrating their biosafety.

**Table 2:** ADMET assets.

	Molecular Weight (g/mol)	Blood- Brain Barrier (BBB)	% Human Intestinal Absorption (HIA +)	TPSA _A_2	logp	nHA	nHD	N rotatable	AMES toxicity	Carcinogenicity
**4**	542.25	NO	98.2	102.25	4.93	9	2	7	Nontoxic	Noncarcinogenic
**5**	466.22	NO	91.3	111.04	4.02	9	3	6	Nontoxic	Noncarcinogenic
**6**	508.23	NO	94.6	119.32	3.24	10	2	7	Nontoxic	Noncarcinogenic
**7**	492.20	NO	75.4	132.40	3.39	10	3	6	Nontoxic	Noncarcinogenic
**8**	508.18	NO	68.3	115.33	4.32	9	3	6	Nontoxic	Noncarcinogenic
**9**	5.18.22	NO	89.8	145.69	3.84	10	4	6	Nontoxic	Noncarcinogenic
**10**	515.22	NO	62.5	149.35	4.27	10	4	6	Nontoxic	Noncarcinogenic
**11**	516.20	NO	80.4	143.30	3.74	10	3	6	Nontoxic	Noncarcinogenic

### Anticancer *in-vitro* study

**Fig. 17:**
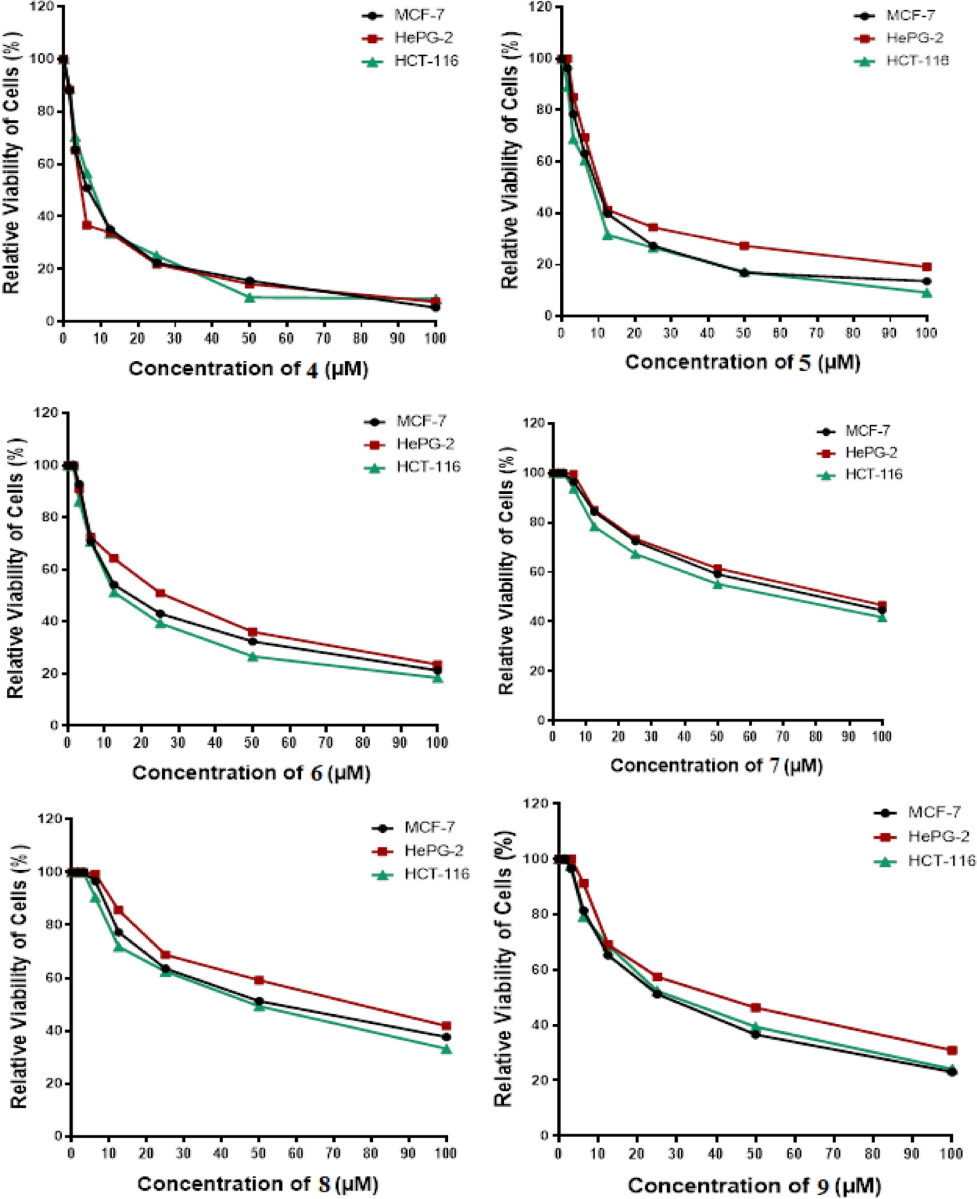

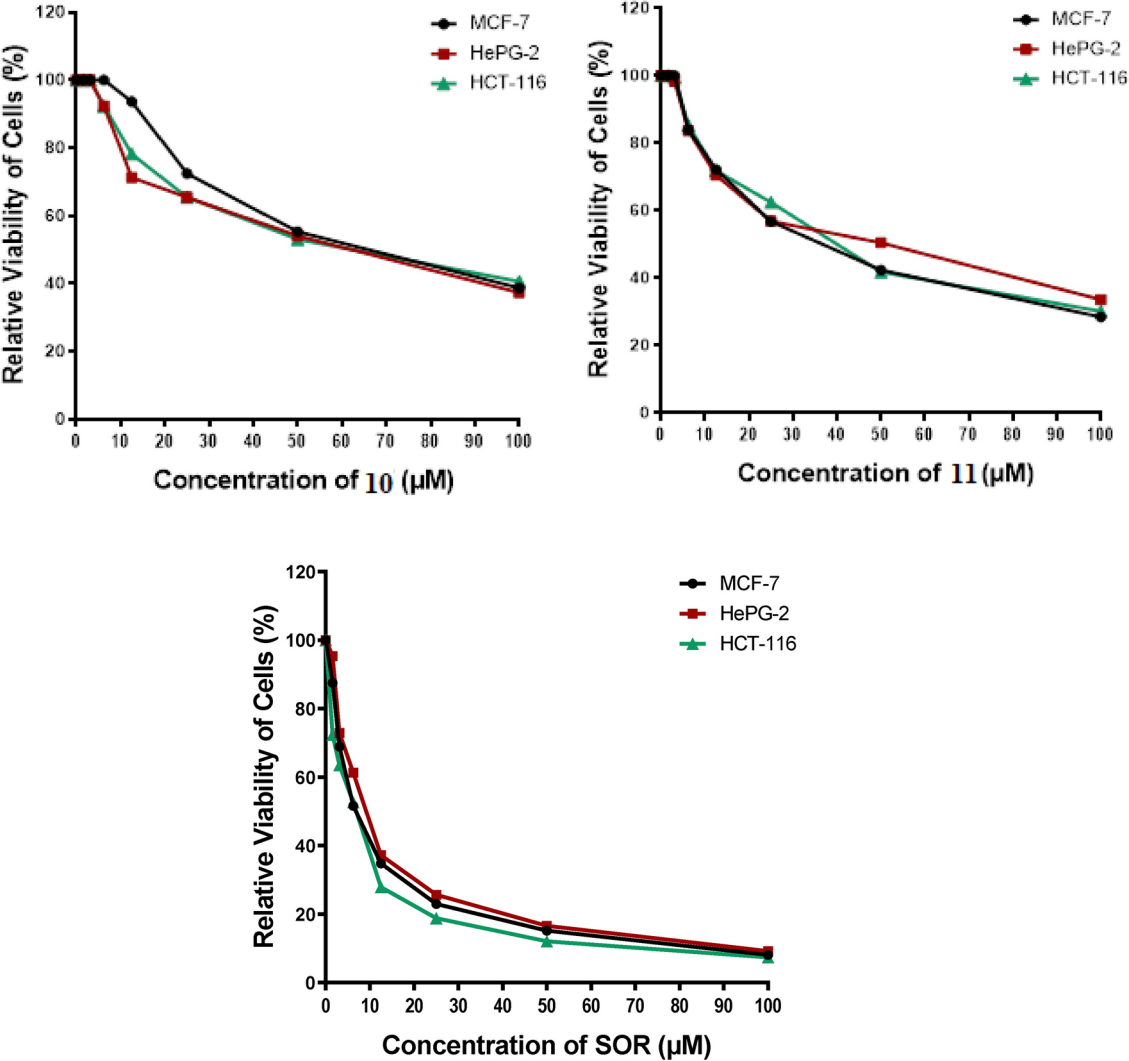
Antitumor activity of compounds **4–11**, against a panel of human tumor cells.

**Fig. 18:**
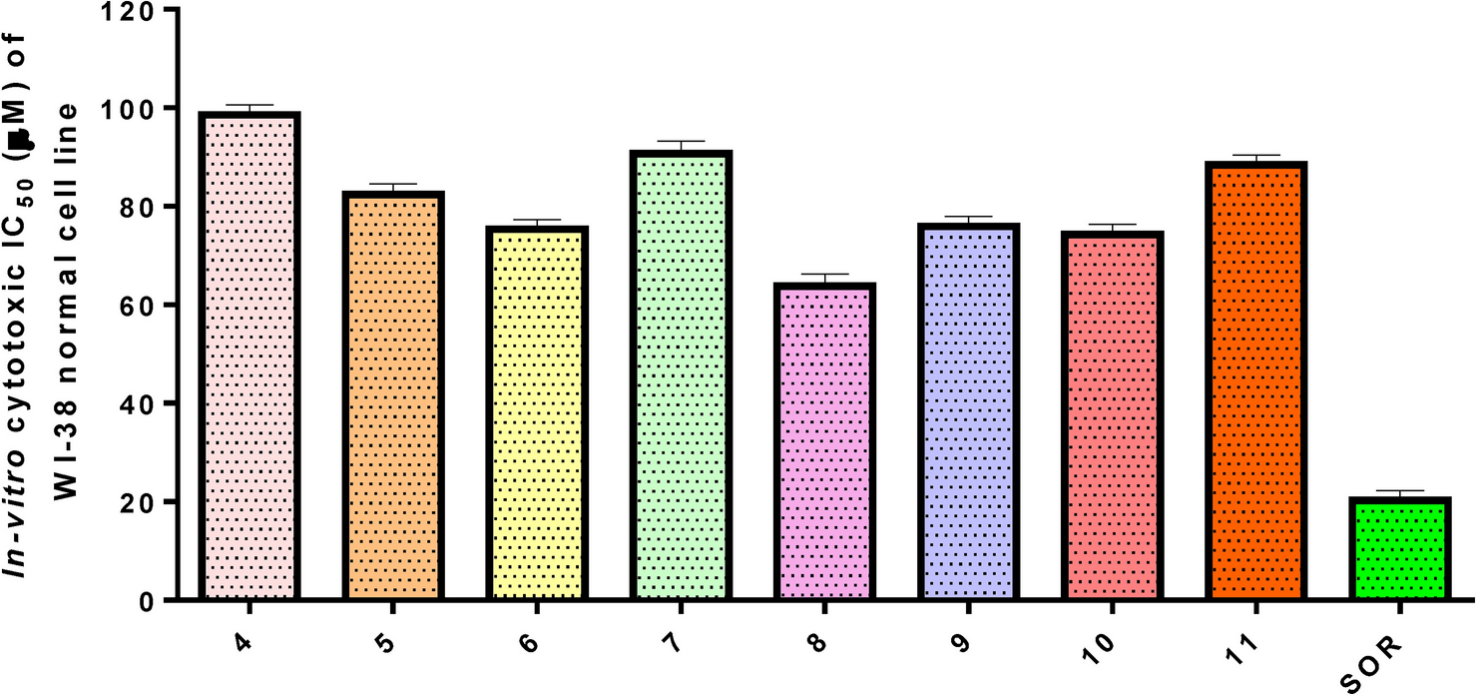
Cytotoxic activity of compounds **4–11**, against human normal WI-38 cell line.

In research on new anticancer agents, the most common experimental screening method after the theoretical study was testing against a group of different cancer cell lines. In this study, an MTT assay was done to determine the antitumor effect of pyrazolone derivatives **(4–11)** compounds on HCT-116, HepG2, and MCF-7 proliferation, and the cytotoxicity limit on WI-38 normal cell line after 48 h, Figs. 17 and 18. Compound **4** showed significant antitumor effects on HCT-116, HepG2, and MCF-7 cancer cell lines with an IC_50_ equal to 7.67 ± 0.5, 5.85 ± 0.4, and 6.97 ± 0.5 μM, respectively. Also, compounds **5** and **6** showed remarkable antitumor effects on HCT-116, HepG2, and MCF-7 cell lines with IC_50_ values (8.14 ± 0.6, 13.98 ± 1.1, 10.34 ± 0.9 μM) (16.53 ± 1.3, 25.62 ± 1.7, 19.81 ± 1.5 μM) respectively. On the other hand, compounds **7**, **8**, **9**, **10,** and **11** showed moderate to weak impact on all panels of cancer cell lines compared with the IC_50_ of Sorafenib (SOR) reference chemotherapeutic drug 5.47 ± 0.3, 9.18 ± 0.6 and 7.26 ± 0.3 μM, respectively. Moreover, all new pyrazolone derivatives **(4–11)** showed lower cytotoxic effects on WI-38 normal cells compared with SOR which observed highly toxic effects on normal cells with IC_50_ equal to 20.27 ± 0.45 μM. This signifies that compound **4**, was effective against proliferative cancer through inhibiting YAP/TEAD mediated Hippo signaling pathway which represented a promising cascade that controls both proliferation and apoptosis. This pathway is crucially regulated by YAP, which does this by shifting its location within the cytoplasm or nucleus. Since YAP lacks a DNA binding domain, its ability to regulate target genes is dependent on its interaction with TEAD^36^. As a result of the newly synthesized pyrazolone derivatives’ potential inhibition of YAP/TEAD target protein, compound **4** could be exploited as promise therapeutic candidate for cancer therapy amoung all synthesized pyrazolones, which by the way confirmed the docking *in-silico* studies.

Our compounds exhibit antioxidant and YAP/TEAD inhibition simultaneously. The suppression of YAP/TEAD targets cancer cells, whereas the antioxidant actions may assist protecting normal cells. Our cell viability investigations have shown anticancer effects, which may be attributed to this dual action. Our compounds’ combination of these qualities’ points to a multi-targeted strategy for treating cancer.

### Structure-anti-cancer activity relationship (SAR)

**Fig. 19:**
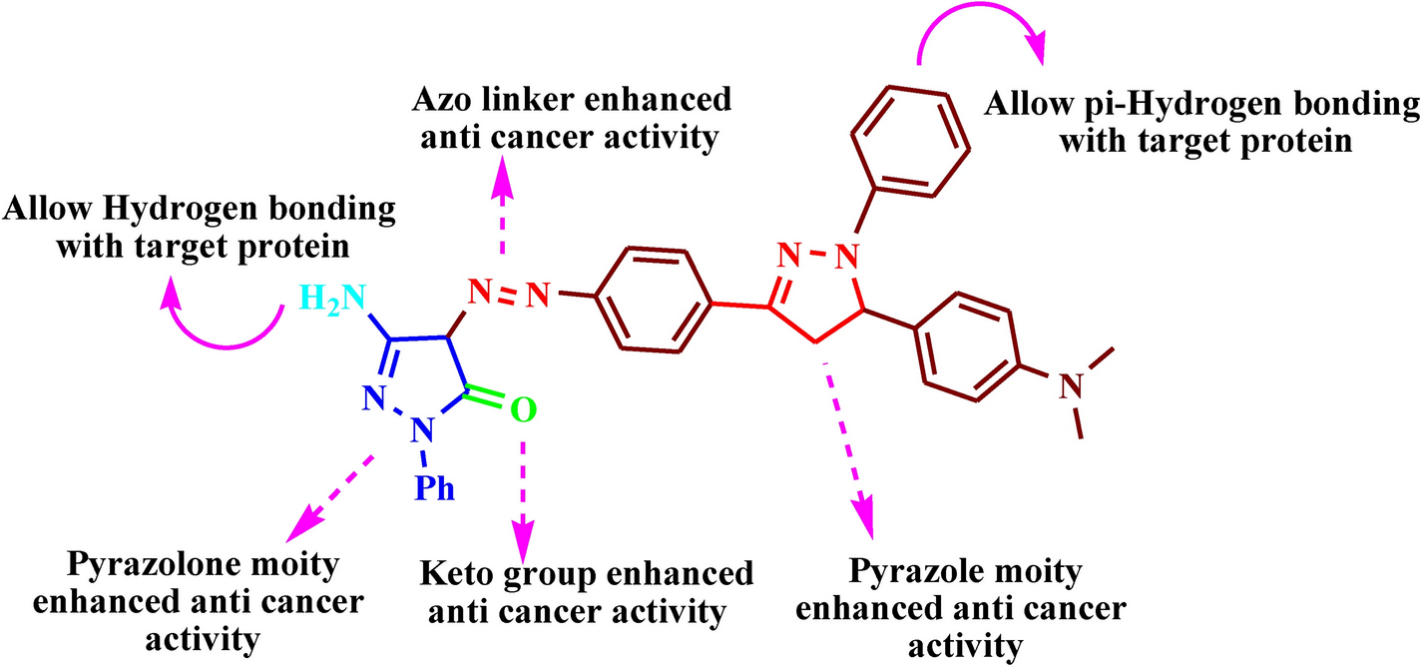
SAR of the most active compound.

Several studies have investigated the structure activity relationship (SAR relationship) of pyrazolones and showed that they have good anti-cancer properties. As illustrated in Fig. 19. The pyrazolone derivatives have a wide range of biological activities due to the presence of different types of interactions with the target YAP/TEAD protein. Firstly, the π-π interaction with the target protein was due to the strong aromaticity of the ring and the presence of heteroatoms like pyrazolone and pyrazole.^37^ secondly, the Hydrogen bonding with the target protein was found due to the presence of both amino group (H. Bond donor) and carbonyl group (H. Bond acceptor).^38^ Finally, some electrostatic attraction forces with the target protein were found due to the presence of azo linker.^39^

## Conclusion

New pyrazolone derivatives (**4–11**) were successfully synthesized and characterized via different spectroscopic techniques. Several important conclusions were obtained from our extensive research.

1. Antioxidant activity; Derivative **4** clarified the most potent antioxidant activity among all other derivatives.
2. Molecular Docking; This *in-silico* study revealed that compound **4**, could be a targeted anticancer agent because it has a good docking score due to hydrogen bonds, and hydrophobic and electrostatic interactions with crucial residues within the binding pocket of the YAP/TEAD target protein.
3. *In-vitro* cytotoxic studies; Confirming the docking inhibitory results, the* in-vitro* anti-cancer activities, against a panel of cancer cell lines were interpreted as the suppressive impact of compound **4** where it triggered apoptosis and obstructed cell survival, growth, via inhibiting of YAP/TEAD mediated Hippo signaling pathway.

Overall, our data identify compound **4** as a new promising candidate for targeted anticancer therapy. Because of its dual role as a potent antioxidant and YAP/TEAD inhibitor, more research is needed to fully investigate its therapeutic potential against various cancer types.

## Supplementary Information

Supplementary Information.

## Data Availability

The cell lines were provided from the American Type Culture Collection (ATCC) via VACSERA, Cairo, Egypt, and all accession codes were added Mammary gland (MCF-7;# *ATCC* HTB-22), colorectal adenocarcinoma (HCT-116; *# **ATCC* CCL-247), hepatocellular carcinoma (HepG-2; #*ATCC* HB-8065), and human lung fibroblast (WI-38; # *ATCC* CCL-75). The datasets generated and/or analyzed during the current study are available in Macromolecule protein structure, and can be deposited in the worldwide protein data bank repository, (https://www.rcsb.org/structure/3KYS)
