# Supplementary material for: Discovering the inhibition of YAP/TEAD Hippo signaling pathway via new pyrazolone derivatives: synthesis, molecular docking and biological investigations
Source: Sci Rep. 2024 Nov 21;14:28859. doi: 10.1038/s41598-024-79992-x (PMC11582634; doi:10.1038/s41598-024-79992-x)
Supplement: Supplementary file 1 — Supplementary Information. [file 41598_2024_79992_MOESM1_ESM.pdf]

Discovering the inhibition of YAP/TEAD Hippo signaling pathway via new pyrazolone derivatives: Synthesis, Molecular docking and Biological investigations

Ahmed A. Noser[1], Maha M. Salem[2], Mohamed H. Baren [1] Adel I Selim [1], and Esraa M ElSafty[1]

S1. Materials and Instrumentations

Unless otherwise stated, all reagents of analytical grade were purchased from Sigma-Aldrich and were used without further purification. All melting points were measured without corrections on a Gallenkamp melting point apparatus. The Fourier transform infrared spectroscopy (FTIR) spectra were recorded on a Perkin-Elmer FTIR 1430 spectrophotometer using KBr disk technique. The ^{1}H nuclear magnetic resonance (NMR) spectra were done at national research center, Cairo, Egypt. It was recorded on a Bruker AC spectrometer (400 MHz) at 25 ^{o}C in DMSO-*d6* with TMS as an internal standard, and chemical shifts are reported in parts per million as d values; ^{13}C NMR was set at 101 MHz. Elemental analyses for C, H and N were also carried out at the Regional Center for Mycology and Biotechnology, Al-Azhar University, and the values were found to be within ±0.4% of the theoretical ones unless otherwise indicated. Reaction progress was monitored by thin-layer chromatography.

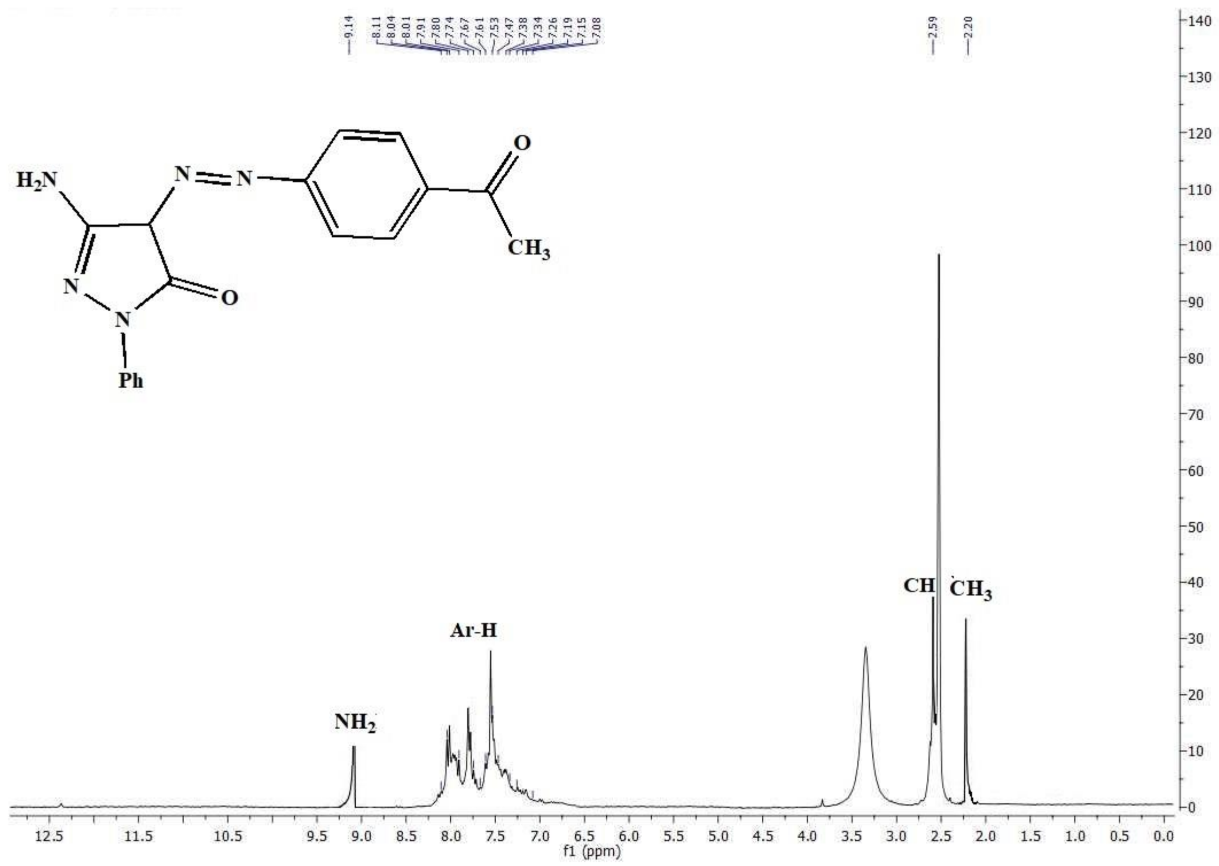

Fig. S1^{1}H-NMR spectrum of **compound 2**

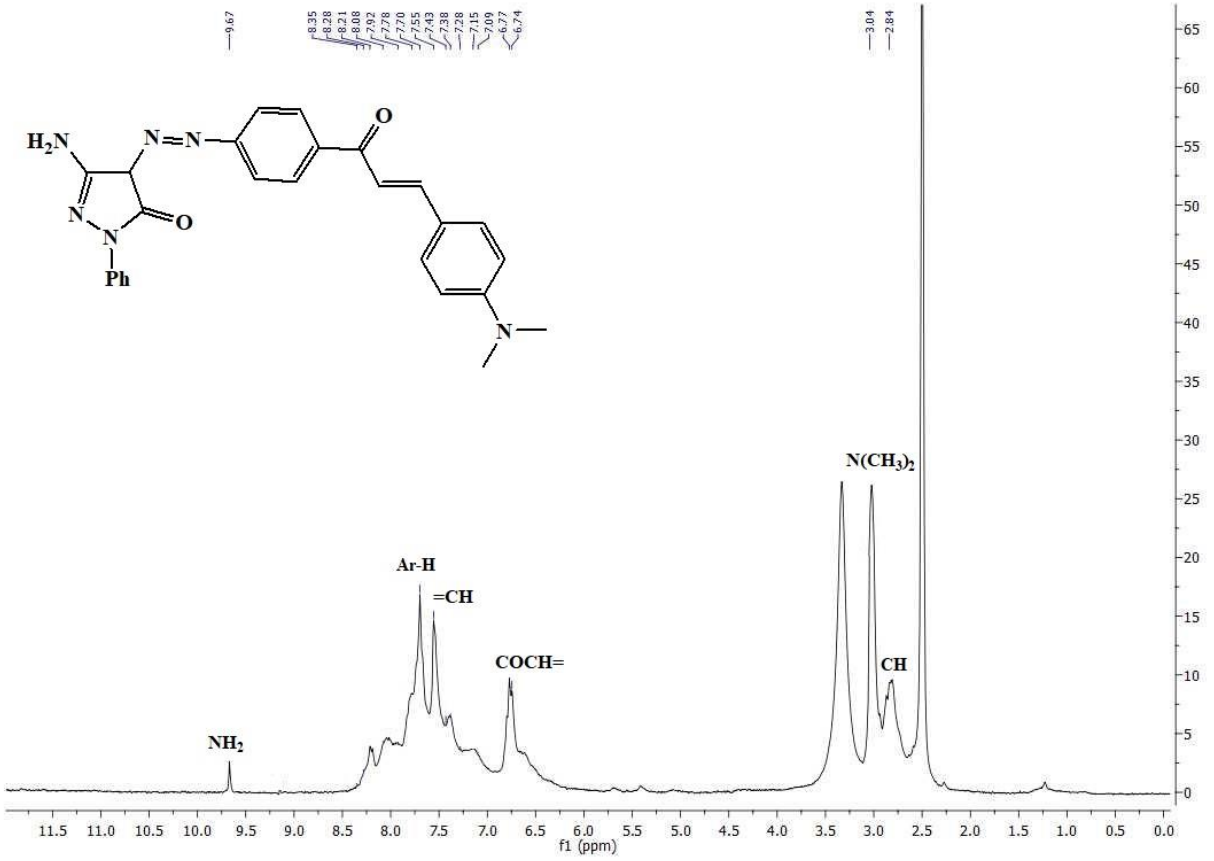

Fig. S2^{1}H-NMR spectrum of **compound 3**

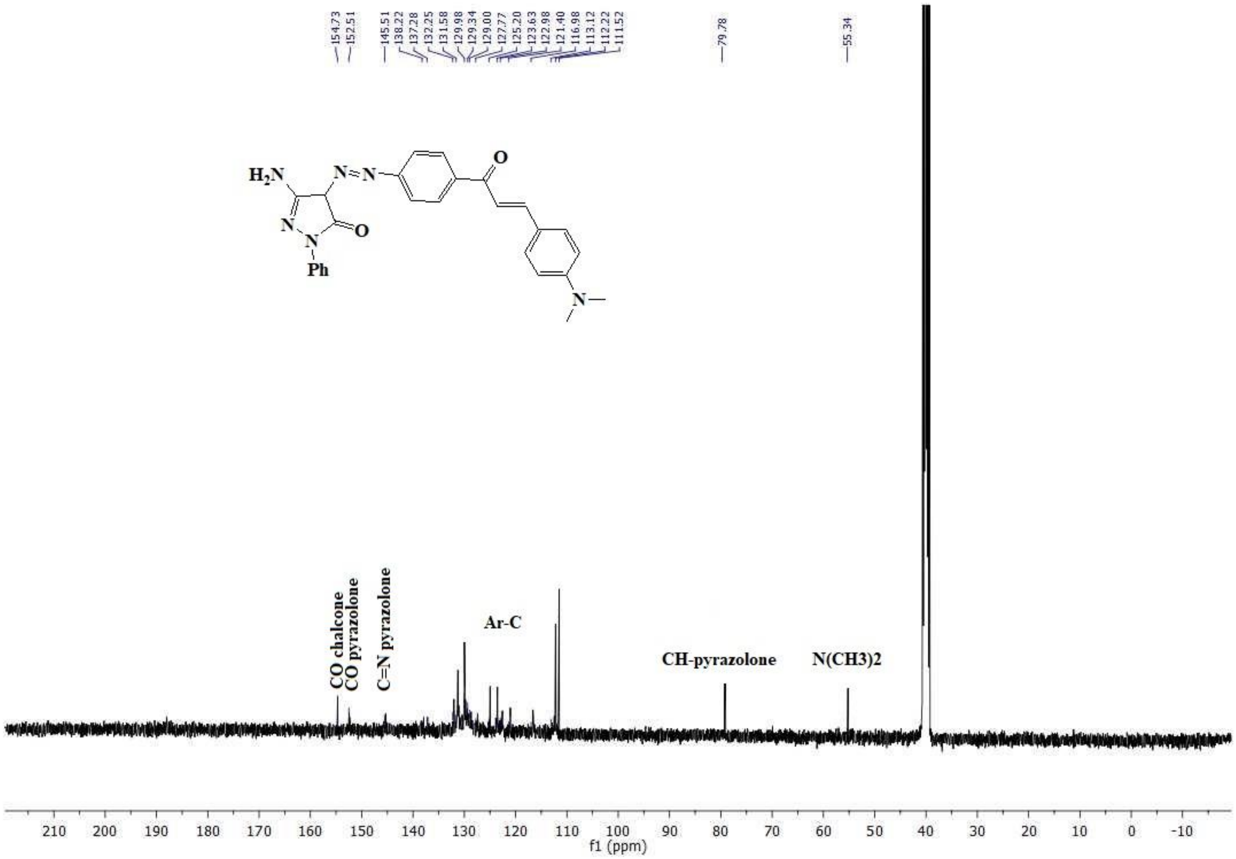

Fig. S3 ^{13}C-NMR spectrum of **compound 3**

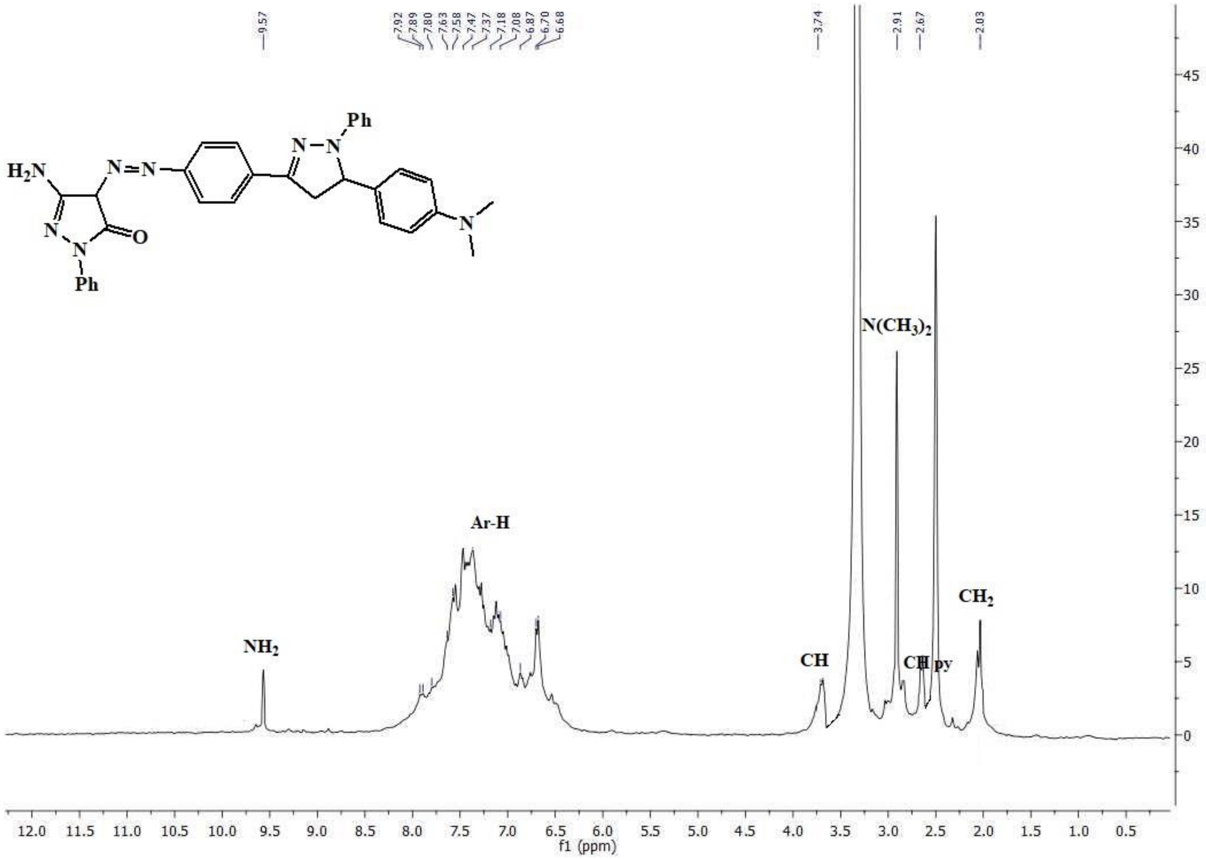

Fig. S4 ^{1}H-NMR spectrum of **compound 4**

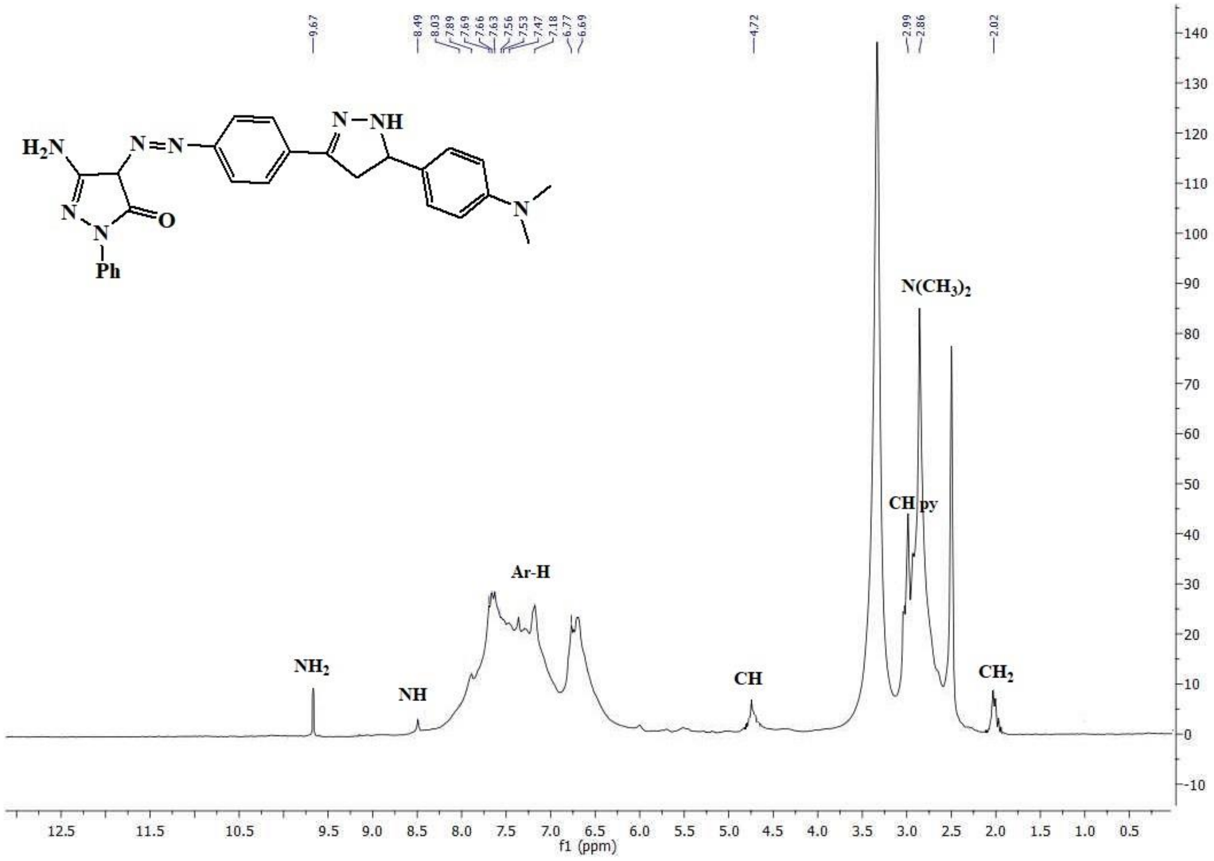

Fig. S5 ^{1}H-NMR spectrum of **compound 5**

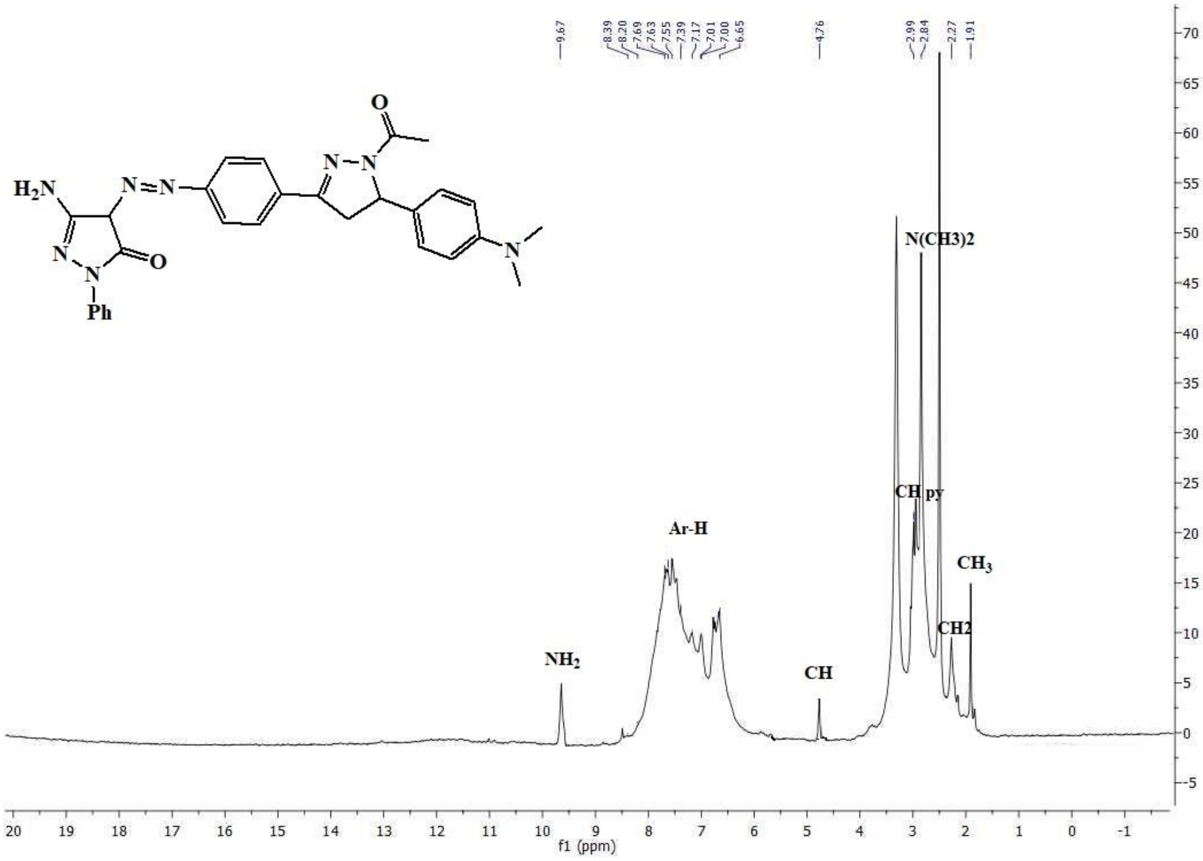

Fig. S6 ^{1}H-NMR spectrum of **compound 6**

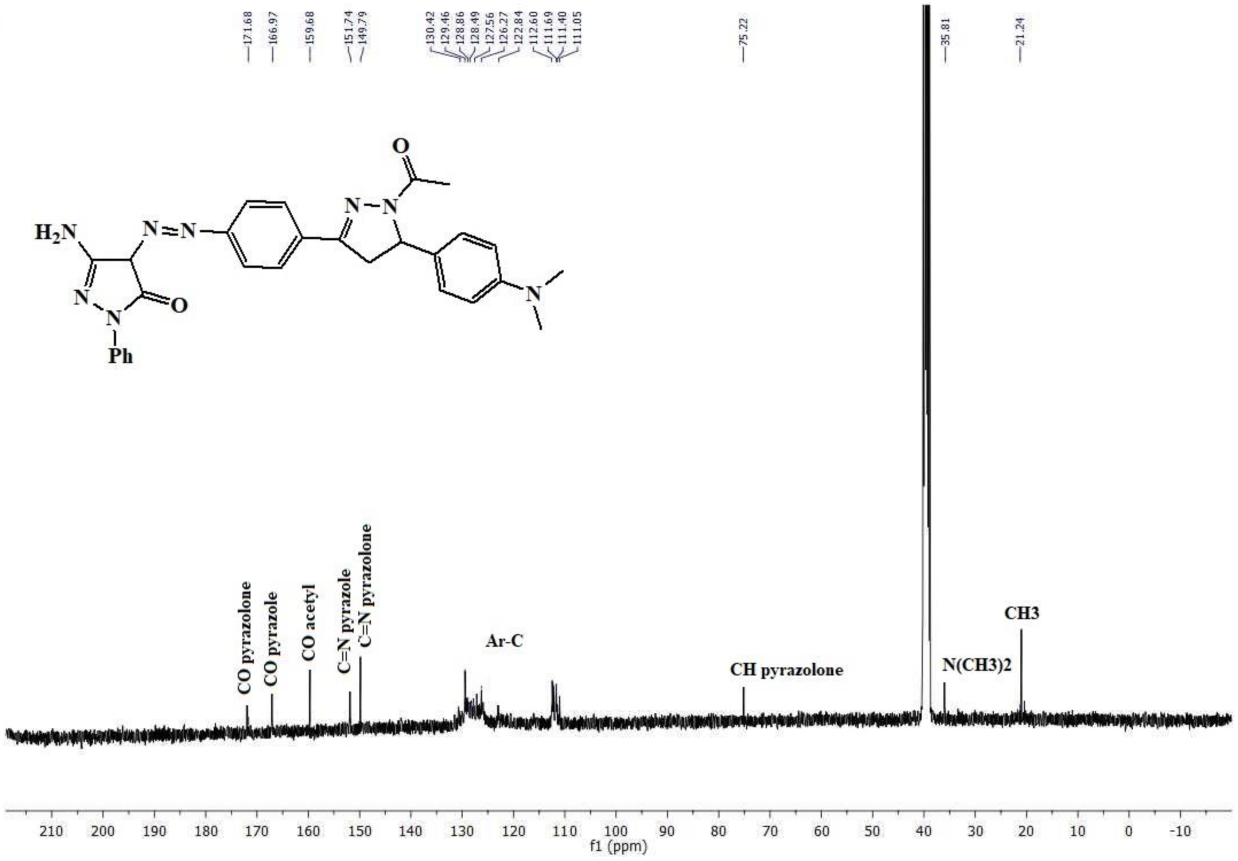

Fig. S7 ^{13}C-NMR spectrum of **compound 6**

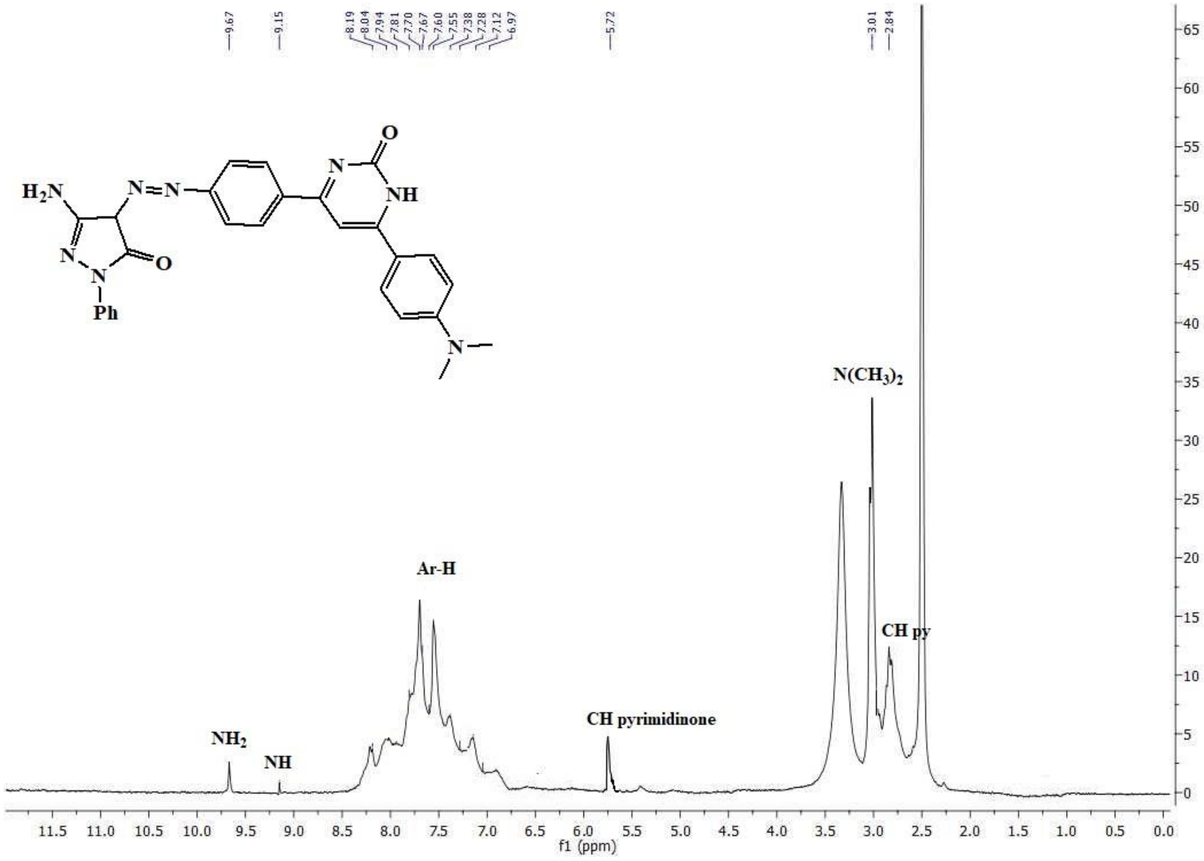

Fig. S8 ^{1}H-NMR spectrum of **compound 7**

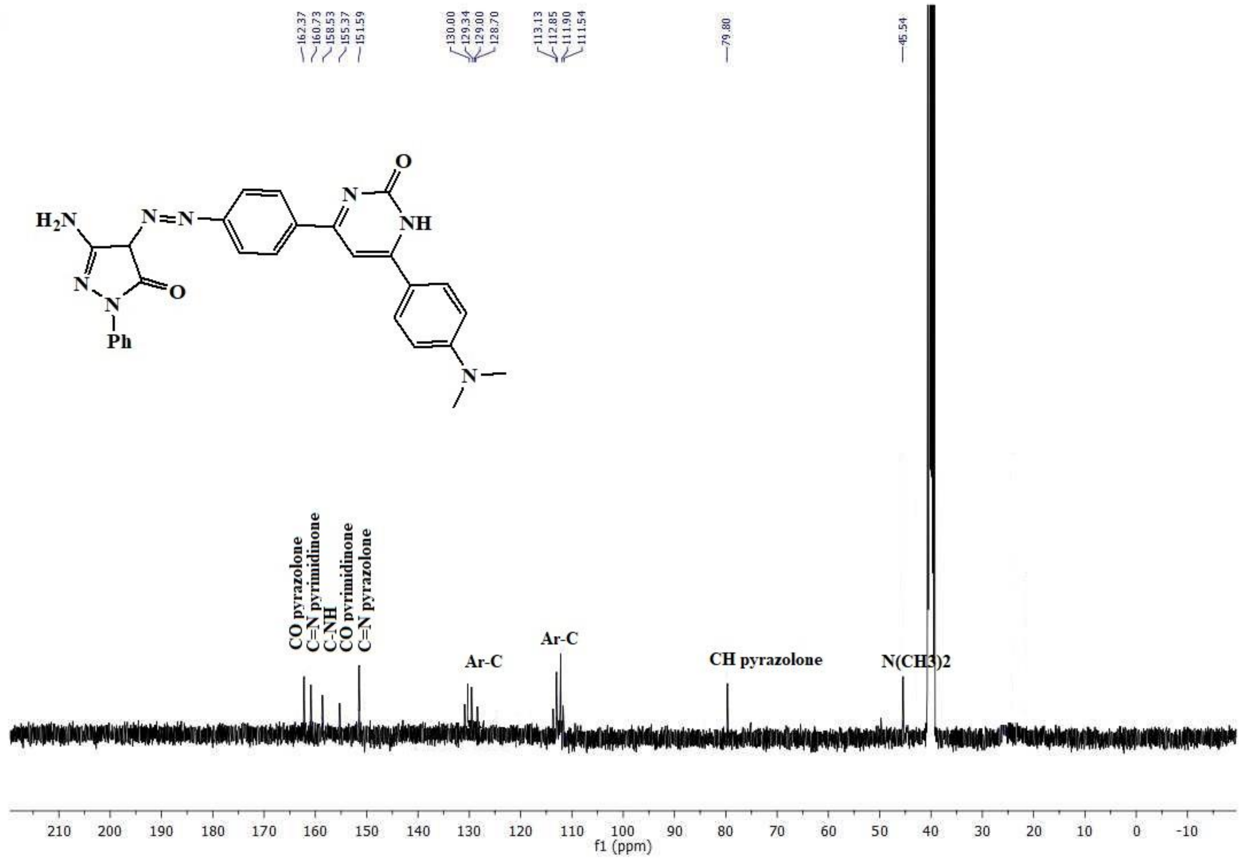

Fig. S9 ^{13}C-NMR spectrum of **compound 7**

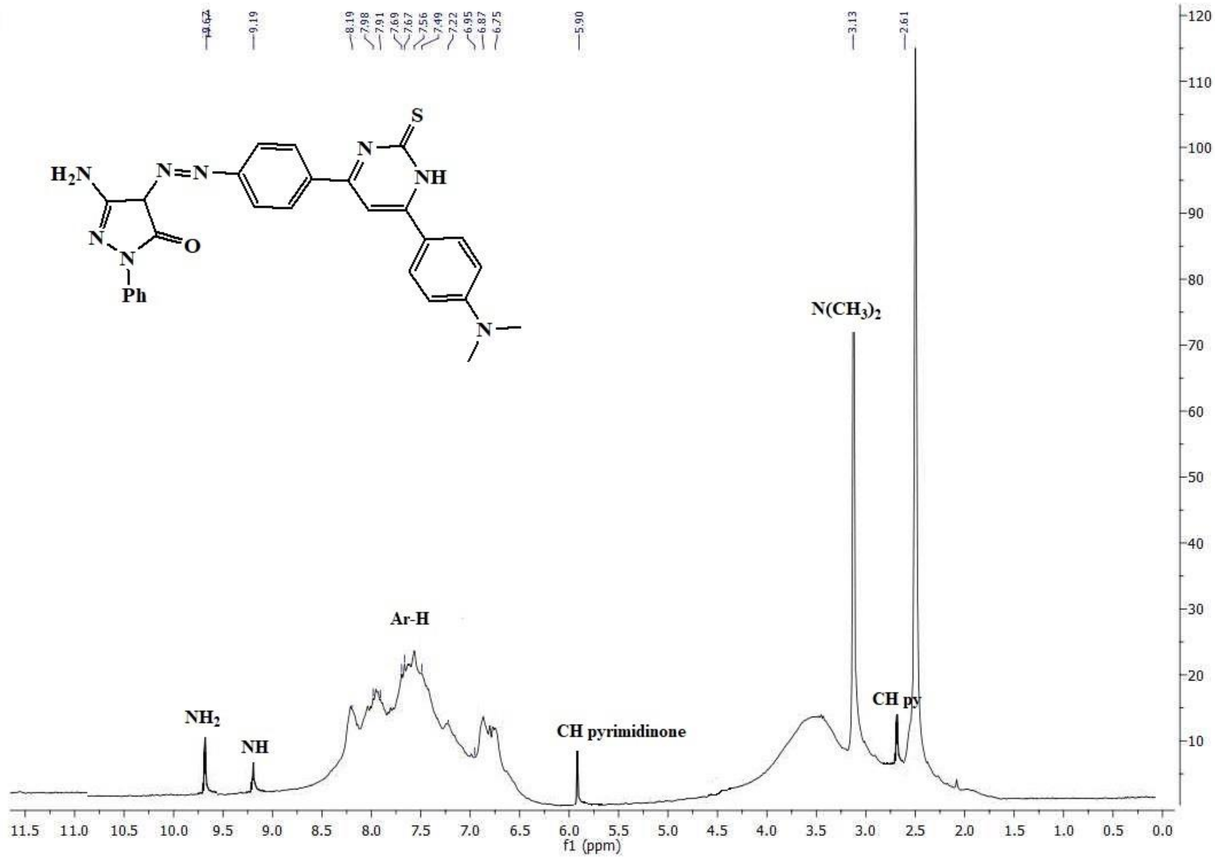

Fig. S10 ^{1}H-NMR spectrum of **compound 8**

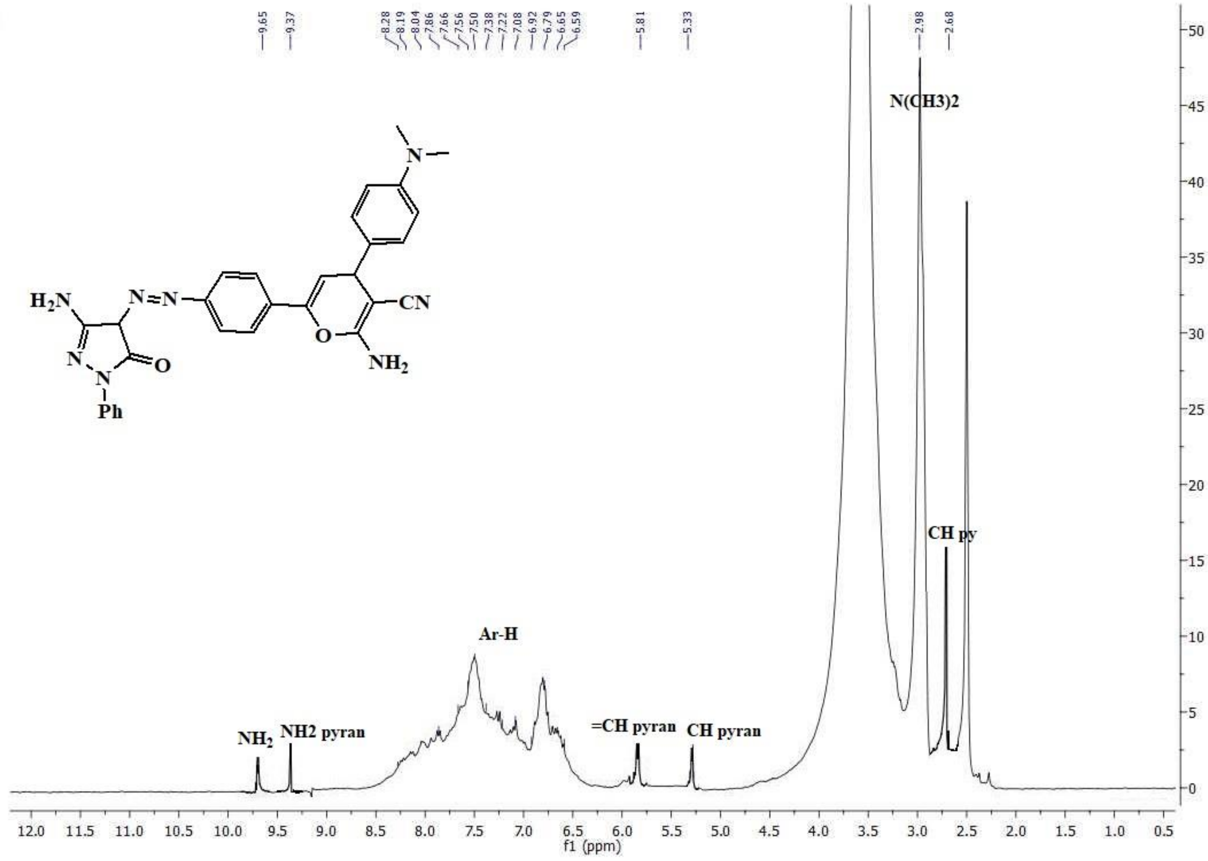

Fig. S11 ^{1}H-NMR spectrum of **compound 9**

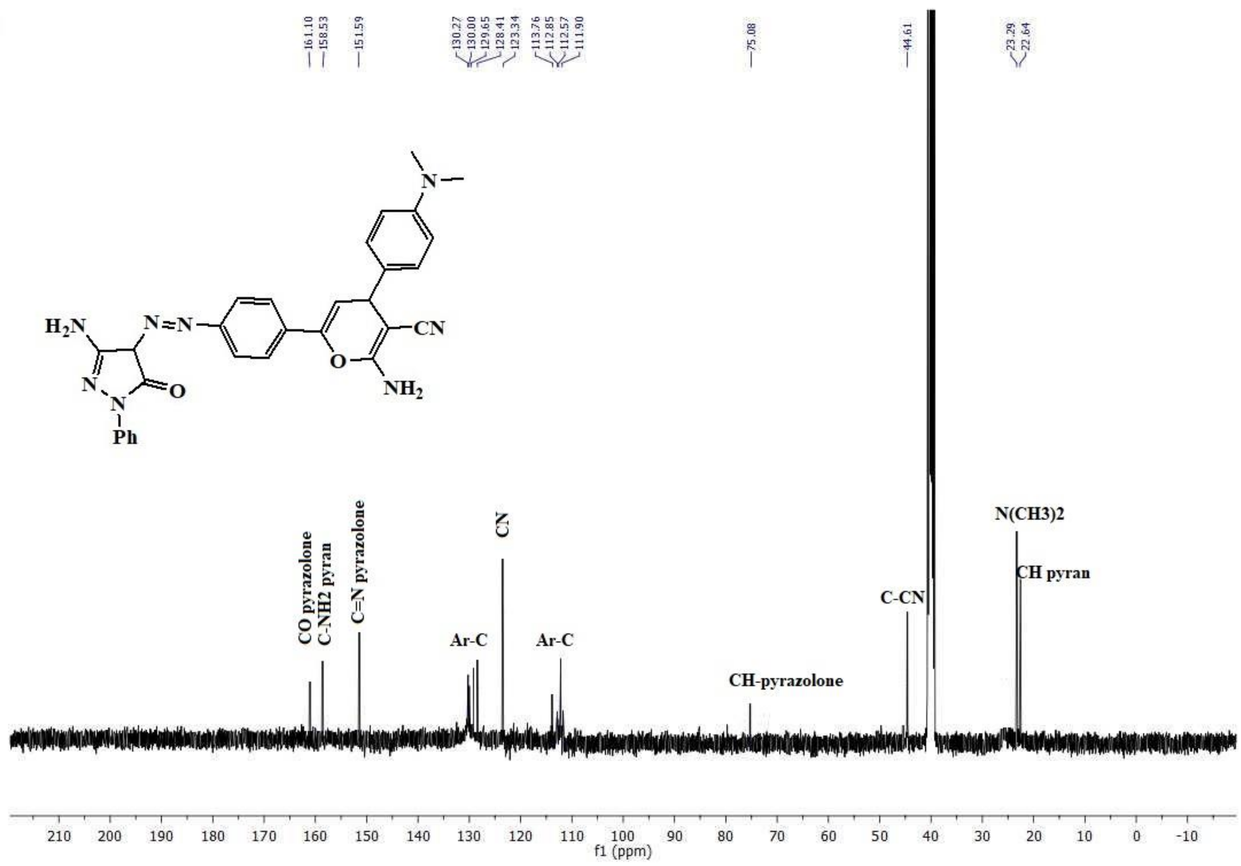

Fig. S12 ^{13}C-NMR spectrum of **compound 9**

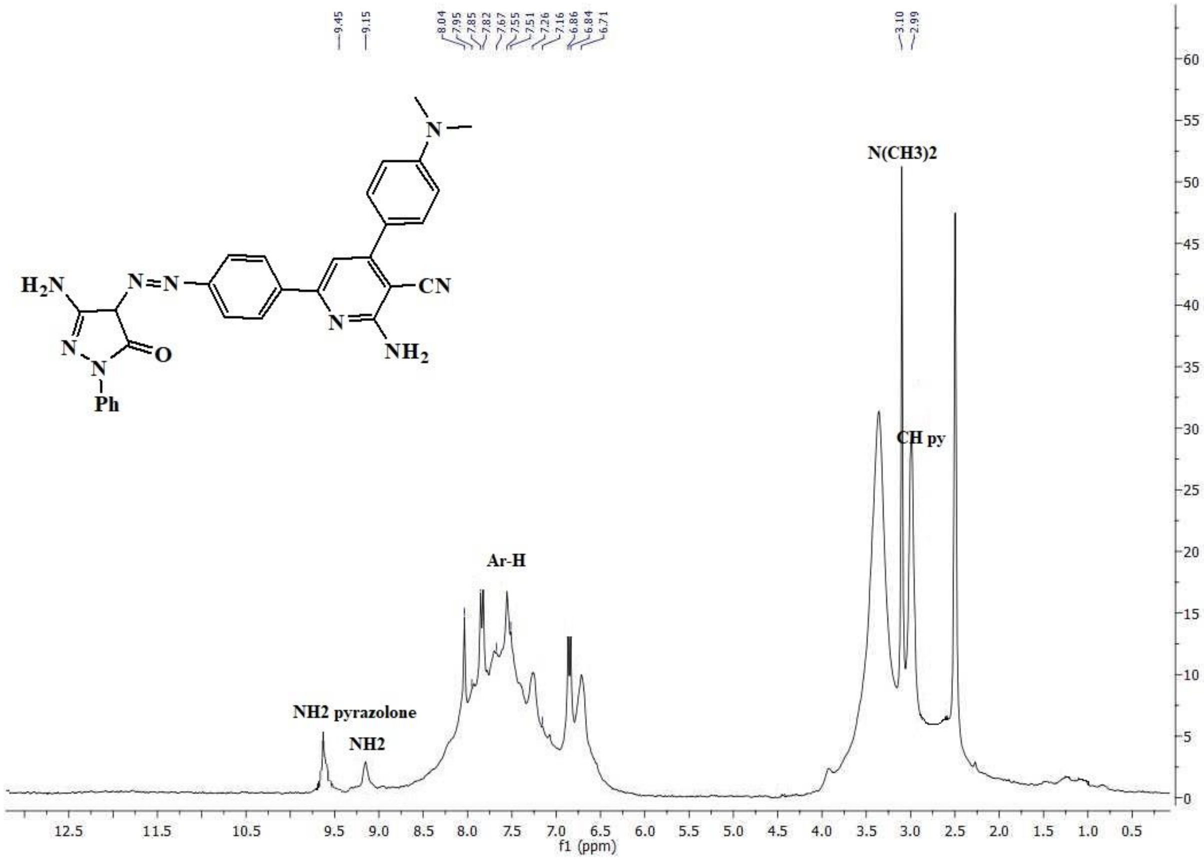

Fig. S13 ^{1}H-NMR spectrum of **compound 10**

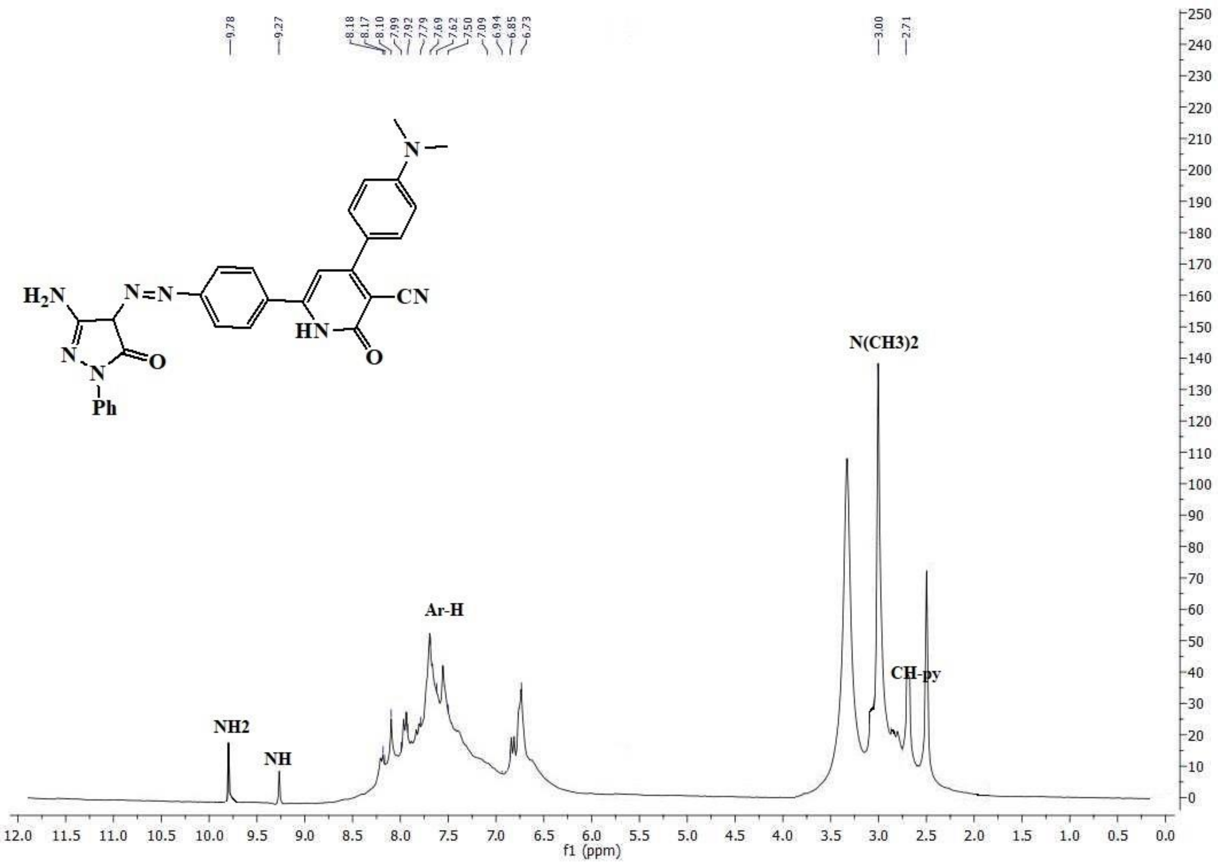

Fig. S14 ^{1}H-NMR spectrum of **compound 11**
